# Integrative phylogenetic analysis of the genus *Episoriculus* (Mammalia: Eulipotyphla: Soricidae)

**DOI:** 10.1371/journal.pone.0299624

**Published:** 2025-01-17

**Authors:** Yingxun Liu, Xuming Wang, Tao Wan, Rui Liao, Shunde Chen, Shaoying Liu, Bisong Yue

**Affiliations:** 1 Sichuan Academy of Forestry, Chengdu, Sichuan, PR China; 2 College of Life Sciences, Sichuan University, Chengdu, Sichuan, PR China; 3 College of Life Sciences, Sichuan Normal University, Chengdu, Sichuan, PR China; Sher-e-Kashmir University of Agricultural Sciences and Technology of Kashmir, INDIA

## Abstract

Shrews in the genus *Episoriculus* are among the least-known mammals in China, where representatives occur mainly in the Himalayan and Hengduan mountains. We sequence one mitochondrial and three nuclear genes from 77 individuals referable to this genus, collect morphometric data for five shape and 11 skull measurements from 56 specimens, and use museum collections and GenBank sequences to analyze phylogenetic relationships between this and related genera in an integrated molecular and morphometric approach. Whereas historically anywhere from two to eight species have been recognized in this genus, we conclude that six (*Episoriculus baileyi*, *E*. *caudatus*, *E*. *leucops*, *E*. *macrurus*, *E*. *sacratus*, *E*. *soluensis*) are valid. We dissent from recent systematic reviews of this genus and regard *E*. *sacratus* to be a valid taxon, *E*. *umbrinus* to be a subspecies of *E*. *caudatus*, and transfer *E*. *fumidus* to *Pseudosoriculus*. Our record of *E*. *soluensis* is the first for China, and expands the previously recognized distribution of this taxon from Nepal and NE India into the adjacent Yadong and Nyalam counties. One further undescribed *Episoriculus* taxon may exist in Xizang.

## Introduction

The genus *Episoriculus*, originally established as a subgenus of *Soriculus*, occurs throughout southwest China, India, Nepal, and Vietnam [1–9]. This subgenus was assigned full generic status by Repenning [10] on grounds of significant differences in tooth morphology from other species of *Chodsigoa* and *Soriculus*—a taxonomy followed by Jameson and Jones [11], Hutterer [12], Wilson and Reeder [13], and Wilson and Mittermeier [3].

The number of valid species of *Episoriculus* has been the subject of debate, with 2–8 species recognized (Table 1). Allen [14] described *Soriculus macrurus*, *S*. *caudatus sacratus*, and *S*. *caudatus umbrinus*. Ellerman and Morrison-Scott [1] proposed *Episoriculu*s as a subgenus of *Soriculus*, and included *S*. *leucops* and *S*. *caudatus* (with subspecies *S*. *c*. *caudatus*, *S*. *c*. *baileyi*, *S*. *c*. *fumidus*, *S*. *c*. *sacratus*, and *S*. *c*. *umbrinus*). Honacki *et al*. [6] proposed that *Episoriculus* included four species, and considered *S*. *baileyi* and *S*. *fumidus* to be valid taxa. Hoffmann [7] similarly recognized four species, although these were not entirely consistent with those of Honacki *et al*. [6], for *S*. *baileyi* was relegated to a subspecies of *S*. (*E*.) *leucops*, and *S*. *(E*.*) macrurus* was placed in this genus. Corbet and Hill [8], and Wilson and Reeder [9, 13] followed this arrangement. Motokawa and Lin [15] elevated *S*. *baileyi* to full species based on morphology. Based on the karyotypes and differences in skull morphology, Motokawa *et al*. [16] considered that *Episoriculus caudatus* should be divided into the larger *E*. *caudatus* and smaller *E*. *sacratus* (with subspecies *E*. *s*. *soluensis* from Nepal and Sikkim, *E*. *s*. *umbrinus* from Assam, Myanmar, and Yunnan, China, and *E*. *s*. *sacratus* from Sichuan, China). He *et al*. [17] noted that *Pseudosoriculus fumidus* did not belong to *Episoriculus*. Based on *CYTB* gene sequences, Abramov *et al*. [2] promoted *E*. *soluensis* to full species, assigned *E*. *fumidus* to a new genus *Pseudosoriculus*, and arranged seven species (*E*. *baileyi*, *E*. *caudatus*, *E*. *leucops*, *E*. *macrurus*, *E*. *sacratus*, *E*. *soluensis*, and *E*. *umbrinus*) in *Episoriculus*. Wilson and Mittermeier [3] recognized eight species, including *P*. *fumidus*.

**Table 1:** Major classification systems of the genus *Episoriculus*. A name of the species in parentheses indicates that the taxon is a subspecies of the previous species.

Allen [14]	Ellerman and Morrison-Scott [1]	Honacki *et al*. [6]	Hoffmann [7]	Wilson and Reeder [9]	Wilson and Reeder [13]	Abramov *et al*. [2]	Wilson and Mittermeier [3]
*S*. *caudatus*	*S*. *caudatus*	*S*. *baileyi*	*S*. *caudatus*	*S*. *caudatus*	*E*. *caudatus*	*E*. *caudatus*	*E*. *caudatus*
*(umbrinus)*	*(umbrinus)*	*S*. *caudatus*	*S*. *leucops*	*S*. *baileyi*	*(sacratus)*	*E*. *umbrinus*	*E*. *umbrinus*
(*sacratus*) *S*. *leucops*	*(baileyi)*	*S*. *fumidus*	*(baileyi)*	*S*. *leucops*	*(umbrinus)*	*E*. *sacratus*	*E*. *sacratus*
	*(sacratus)*	*S*. *leucops*	*S*. *macrurus*	*S*. *macrurus*	*E*. *fumidus*	*E*. *leucops*	*E*. *leucops*
	*(fumidus)*		*S*. *fumidus*	*S*. *fumidus*	*E*. *macrurus*	*E*. *macrurus*	*E*. *macrurus*
	*S*. *leucops*				*E*. *leucops*	*E*. *soluensis*	*E*. *soluensis*
					*(baileyi)*	*E*. *baileyi*	*E*. *fumidus*
							*E*. *baileyi*

Throughout these various classifications the taxonomic status of *E*. *caudatus*, *E*. *leucops*, and *E*. *macrurus* has been relatively stable, but the taxonomy of *P*. *fumidus*, *E*. *sacratus*, E. *umbrinus*, *E*. *baileyi*, and *E*. *soluensis* has not. We report new molecular data and morphological comparisons in an integrated phylogenetic and morphological analysis to clarify the taxonomic status of species in the genus *Episoriculus*.

## Materials and methods

### Field methodology

In the study, traplines were installed to capture shrews. We use red plastic buckets as traps. Each of these 7L buckets was 22 cm high and had an upper and lower diameter of 26 cm and 19 cm, respectively. We typically position these barrels in the more humid forest, primarily around the roots of massive, or falling trees, 3-5m apart. The buckets were often put in the afternoon of the first day and checked the following morning to see whether any shrews have fallen into the trap. The time between placement and inspection is assured to be more than 12 hours.

After euthanasia with eugenol, following the ASM guidelines [18], tissue samples were taken from the thigh muscle and stored in absolute ethyl alcohol at ambient temperature. Voucher specimens fixed in 10% formalin before transferal to 95% ethanol for long term preservation and deposition of vouchers at the Zoological Museum of Sichuan Academy of Forestry (SAF), Chengdu, Sichuan, China. All specimens were collected in accordance with regulations in China for implementation of the protection of terrestrial wild animals (State Council Decree [1992] No. 13). Collecting protocols and the research project were approved by the Ethics Committee of Sichuan Academy of Forestry (2024–001).

### Sampling and sequencing

**Fig 1:**
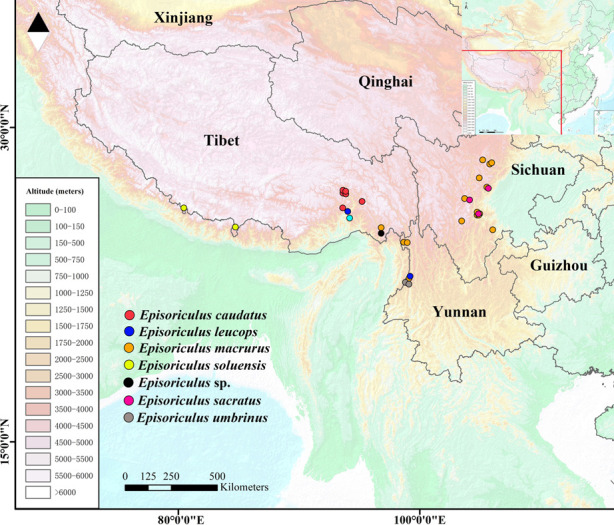
Map of genus *Episoriculus*, showing localities sampled for this study. Reprinted from Fan *et al*. (2022) [Doi: https://doi.org/10.1002/ece3.9404] under a CC BY license, with permission from Fan Ronghui, original copyright 2022. And edited and supplemented by Adobe Photoshop 2022 and Arcgis 10.8.

All specimens were identified morphologically following the original literature species established [19–23], and some subsequent studies on this group, such as Wilson and Mittermeier [3], Hoffmann [7], and Smith and Xie [24] in which, some specimens which were be identified as *E*. *sacratus* and *E*. *soluensis*, were collected adjacent of the type locality (Table 2). For verifying our species accuracies, some sequences collected form type locality or adjacent of the type locality, were downloaded [2, 17, 25–32] (Table 3). Of recognized species, we had no specimens or sequences of *E*. *baileyi*. Through preliminary molecular and morphological identification, 77 specimens collected from China, 31 were attributed to *E*. *macrurus*, 18 to *E*. *caudatus*, 10 to *E*. *leucops*, 5 to *E*. *umbrinus*, 4 of each to *E*. *sacratus* and *E*. *soluensis*, and 2 to *E*. sp. (Table 1 and Fig 1)

**Table 2:** Samples and sequences of *Episoriculus* used for molecular analyses.

Species	Species ID	Sequence No.	Locality	Relative location	longitude	Latitude	Altitude(m)	Genbank accession No.			
								*CYTB*	*APOB*	*BRCA1*	*RAG2*
*Episoriculus sacratus*	SAF15216	LS15073	Lushan, Sichuan		103.04592	30.66332	2018	MK962202	/	MN032211	MN032279
*Episoriculus sacratus*	SAF06888	ELSTNPB01007	Erlangshan, Sichuan	adjacent to type locality	102.29916	29.88547	2450	MK962204	MN032147	MN032213	MN032281
*Episoriculus sacratus*	SAF06902	ELSTNPB02004	Erlangshan, Sichuan	adjacent to type locality	102.29916	29.88547	2450	MK962207	MN032149	MN032214	MN032283
*Episoriculus sacratus*	SAF11594	BLG012	Balanggou, Sichuan	adjacent to type locality	101.98069	30.41007	2460	MK962218	/	MN032223	/
*Episoriculus sacratus*	SAF06934	ELS260B02003	Erlangshan, Sichuan	adjacent to type locality	102.31112	29.87314	2230	MK962203	/	MN032212	MN032280
*Episoriculus sacratus*	SAF06920	ELS260A02003	Erlangshan, Sichuan	adjacent to type locality	102.30982	29.87294	2220	MK962217	/	MN032222	MN032292
*Episoriculus umbrinus*	SAF13199	NJ13033	Lushui, Yunnan		98.70969	25.98614	2719	MK962237	MN032174	MN032241	MN032310
*Episoriculus umbrinus*	SAF13257	NJ13091	Gongshan, Yunnan		98.44691	27.77396	3320	MK962198	/	MN032207	/
*Episoriculus umbrinus*	SAF180044	YN18021	Mountain Gaoligong, Yunnan		98.7111	25.97795	2570	MK962223	MN032163	MN032228	MN032297
*Episoriculus caudatus*	SAF07474	XZRAP05005	Nyingchi, Xizang		94.962862	30.00965	2040	MK962213	MN032155	MN032218	MN032289
*Episoriculus caudatus*	SAF07492	XZRAP07004	Nyingchi, Xizang		94.9631	30.00942	2020	MK962212	MN032154	MN032217	MN032288
*Episoriculus caudatus*	SAF07568	XZXCY04003	Xiachayu, Xizang		97.01193	28.51268	1640	MK962224	/	MN032229	MN032298
*Episoriculus caudatus*	SAF07483	XZRAP06001	Nyingchi, Xizang		94.95855	30.00766	2050	MK962214	MN032156	MN032219	MN032290
*Episoriculus caudatus*	SAF07566	XZXCY04001	Xiachayu, Xizang		97.01193	28.51268	1640	MK962206	/	/	/
*Episoriculus caudatus*	SAF07502	XZRAP08004	Nyingchi, Xizang		94.95878	30.01025	2040	MK962219	MN032159	MN032224	MN032293
*Episoriculus caudatus*	SAF07501	XZRAP08003	Nyingchi, Xizang		94.95878	30.01025	2040	MK962222	MN032162	MN032227	MN032296
*Episoriculus caudatus*	SAF07472	XZRAP05003	Nyingchi, Xizang		94.962862	30.00965	2040	MK962221	MN032161	MN032226	MN032295
*Episoriculus caudatus*	SAF07490	XZRAP07002	Nyingchi, Xizang		94.9631	30.00942	2020	MK962215	MN032157	MN032220	/
*Episoriculus caudatus*	SAF07535	XZLZT02001	Nyingchi, Xizang		95.00633	30.02289	2020	MK962211	MN032153	/	MN032287
*Episoriculus caudatus*	SAF07470	XZRAP05001	Nyingchi, Xizang		94.962862	30.00965	2040	MK962216	MN032158	MN032221	MN032291
*Episoriculus* sp.	SAF11247	MT11162	Motuo, Xizang		95.4814	29.43014	2832	MK962200	MN032145	MN032209	MN032277
*Episoriculus* sp.	SAF11283	MT11198	Motuo, Xizang		95.0095	29.40422	3118	MK962199	MN032144	MN032208	MN032276
*Episoriculus leucops*	SAF180058	YN18035	Mountain Gaoligong, Yunnan		98.7145	25.95919	2250	MK962232	MN032169	MN032237	MN032306
*Episoriculus leucops*	SAF180035	YN18012	Mountain Gaoligong, Yunnan		98.7111	25.97795	2570	MK962231	MN032168		MN032305
*Episoriculus leucops*	SAF13190	NJ13024	Lushui, Yunnan		98.68388	25.97259	3150	MK962234	MN032171	MN032239	MN032308
*Episoriculus leucops*	SAF180036	YN18013	Mountain Gaoligong, Yunnan		98.7111	25.97795	2570	MK962227	MN032165	MN032232	MN032301
*Episoriculus leucops*	SAF180033	YN18010	Mountain Gaoligong, Yunnan		98.7111	25.97795	2570	MK962229	MN032167	MN032234	MN032303
*Episoriculus leucops*	SAF180034	YN18011	Mountain Gaoligong, Yunnan		98.7111	25.97795	2570	MK962233	MN032170	MN032238	MN032307
*Episoriculus leucops*	SAF180045	YN18022	Mountain Gaoligong, Yunnan		98.7111	25.97795	2570	MK962226	/	MN032231	MN032300
*Episoriculus leucops*	SAF180040	YN18017	Mountain Gaoligong, Yunnan		98.7111	25.97795	2570	MK962228	MN032166	MN032233	MN032302
*Episoriculus leucops*	SAF180037	YN18014	Mountain Gaoligong, Yunnan		98.7111	25.97795	2570	MK962230	/	MN032235	MN032304
*Episoriculus leucops*	SAF11202	MT11017	Motuo, Xizang		95.126722	29.3665	2100	MK962235	MN032172	MN032240	MN032309
*Episoriculus macrurus*	SAF180039	YN18016	Mountain Gaoligong, Yunnan		98.7111	25.97795	2570	MK962166	/	MN032180	MN032247
*Episoriculus macrurus*	SAF13198	NJ13032	Lushui, Yunnan		98.70969	25.98614	2719	MK962170	MN032126	MN032184	MN032251
*Episoriculus macrurus*	SAF13256	NJ13090	Gongshan, Yunnan		98.44691	27.77396	3320	MK962165	/	MN032179	MN032246
*Episoriculus macrurus*	SAF13288	NJ13122	Gongshan, Yunnan		98.50351	27.79702	3050	MK962167	/	MN032181	MN032248
*Episoriculus macrurus*	SAF13197	NJ13031	Lushui, Yunnan		98.70969	25.98614	2719	MK962163	/	MN032177	/
*Episoriculus macrurus*	SAF07587	CY17	Chayu, Xizang		97.01826	28.77404	2900	MK962236	MN032173	/	/
*Episoriculus macrurus*	SAF09629	JJSA506	Jinjiashan, Sichuan		102.75222	30.84041	2620	MK962195	/	MN032205	MN032274
*Episoriculus macrurus*	SAF181173	WL18246	Wanglang, Sichuan		104.1342	32.94794	2570	MK962179	MN032134	/	MN032260
*Episoriculus macrurus*	SAF06900	ELSTNPB02002	Erlangshan, Sichuan		102.29916	29.88547	2450	MK962175	MN032131	MN032188	MN032256
*Episoriculus macrurus*	SAF06829	ELSMYPA02004	Erlangshan, Sichuan		102.28433	29.86774	2780	MK962162	/	MN032176	MN032244
*Episoriculus macrurus*	SAF11599	BLG031	Balanggou, Sichuan		101.91481	30.40052	2820	MK962187	MN032139	MN032197	/
*Episoriculus macrurus*	SAF06932	ELS260B02001	Erlangshan, Sichuan		102.31112	29.87314	2230	MK962173	/	MN032187	MN032254
*Episoriculus macrurus*	SAF06874	ELSTNPA02002	Erlangshan, Sichuan		102.29953	29.88633	2430	MK962176	/	MN032189	MN032257
*Episoriculus macrurus*	SAF181086	WL18159	Wanglang, Sichuan		104.1437	32.928634	2500	MK962180	MN032135	/	MN032261
*Episoriculus macrurus*	SAF181215	WL18288	Wanglang, Sichuan		117.1342	45.94794	2583	MK962183	/	MN032193	MN032263
*Episoriculus macrurus*	SAF181127	WL18200	Wanglang, Sichuan		104.1459	32.927797	2500	MK962189	MN032140	MN032199	MN032268
*Episoriculus macrurus*	SAF181126	WL18199	Wanglang, Sichuan		104.1459	32.927797	2500	MK962188	/	MN032198	MN032267
*Episoriculus macrurus*	SAF181065	WL18138	Wanglang, Sichuan		104.1437	32.928634	2500	MK962185	/	MN032195	MN032265
*Episoriculus macrurus*	SAF181130	WL18203	Wanglang, Sichuan		104.1459	32.927797	2500	MK962184	MK962184	MN032194	MN032264
*Episoriculus macrurus*	SAF181129	WL18202	Wanglang, Sichuan		104.1459	32.927797	2500	MK962190	MN032141	MN032200	MN032269
*Episoriculus macrurus*	SAF09655	JJSA532	Balanggou, Sichuan		102.75311	30.84053	2620	MK962193	MN032143	MN032203	MN032272
*Episoriculus macrurus*	SAF181137	WL18137	Wanglang, Sichuan		104.1459	32.927797	2500	MK962182	MN032136	MN032192	/
*Episoriculus macrurus*	SAF06725	ELSLCA03001	Erlangshan, Sichuan		102.26703	29.85678	2800	MK962172	/	MN032186	MN032253
*Episoriculus macrurus*	SAF181394	WL18467	Wanglang, Sichuan		103.9963	32.96181	3000	MK962174	MN032130	/	MN032255
*Episoriculus macrurus*	SAF181128	WL18201	Wanglang, Sichuan		104.1459	32.927797	2500	MK962181	/	MN032191	MN032262
*Episoriculus macrurus*	SAF15165	LS15023	Lushan, Sichuan		103.01808	30.68135	2424	MK962194	/	MN032204	MN032273
*Episoriculus macrurus*	SAF06730	ELSDGA01003	Erlangshan, Sichuan		102.2584	29.85416	2700	MK962196	/	MN032206	MN032275
*Episoriculus macrurus*	SAF06767	ELSBTDBS01013	Erlangshan, Sichuan		102.26283	29.85202	2500	MK962161	/	MN032175	MN032243
*Episoriculus macrurus*	JJSA137	JJSA137	Jiajinshan, Sichuan					MK962192	MN032142	MN032202	MN032271
*Episoriculus macrurus*	SAF181202	WL18275	Wanglang, Sichuan		104.1342	32.94794	2570	MK962178	MN032133	/	MN032259
*Episoriculus macrurus*	SAF09303	JJSA443	Jiajinshan, Sichuan		10268985	30.84276	3440	MK962186	MN032138	MN032196	MN032266
*Episoriculus macrurus*	HLG01017	HLG01017	Hailuogou, Sichuan					MK962191	/	MN032201	MN032270
*Episoriculus macrurus*	SAF06177	MGDW0502	Meigu, Sichuan		103.27607	28.7063	2900	MK962177	MN032132	MN032190	MN032258
*Episoriculus soluensis*	SAF13503	XZ13041	Nyalam, Xizang	adjacent to type locality	85.99854	28.0815	3200	MK962201	MN032146	MN032210	MN032278
*Episoriculus soluensis*	SAF14536	XZ14002	Yadong, Xizang		88.994744	27.540042	4304	MK962157	/	/	/
*Episoriculus soluensis*	SAF14537	XZ14003	Yadong, Xizang		88.994744	27.540042	4304	MK962158	/	/	/
*Episoriculus soluensis*	SAF14538	XZ14004	Yadong, Xizang		88.994744	27.540042	4304	MK962159	/	/	/

**Table 3:** GenBank accession numbers of download sequence from NCBI.

Species	Locality	Relative location	*CYTB*	*APOB*	*BRCA1*	*RAG2*	Cited source
*Anourosorex squamipes*	Baoxing, Sichuan, China	type locality	KT032922	/	/	/	He *et al*. [25]
*Anourosorex squamipes*	Baoxing, Sichuan, China	type locality	KT032921	/	/	/	He *et al*. [25]
*Anourosorex squamipes*	Baoxing, Sichuan, China	type locality	KT032920	/	/	/	He *et al*. [25]
*Anourosorex squamipes*	Baoxing, Sichuan, China	type locality	KT032919	/	/	/	He *et al*. [25]
*Blarinella griselda*	Yunnan, China		GU981259	GU981109	GU981184	/	He *et al*. [17]
*Blarinella griselda*	Yunnan, China		GU981258	GU981108	GU981183	GU981441	He *et al*. [17]
*Blarinella quadraticauda*	Baoxing, Sichuan, China	type locality	JF719718	/	/	/	Chen *et al*. [26]
*Blarinella quadraticauda*	Baoxing, Sichuan, China	type locality	JF719719	/	/	/	Chen *et al*. [26]
*Blarinella quadraticauda*	Baoxing, Sichuan, China	type locality	JF719720	/	/	/	Chen *et al*. [26]
*Blarinella quadraticauda*	Baoxing, Sichuan, China	type locality	JF719721	/	/	/	Chen *et al*. [26]
*Chimarrogale himalayica*	Yunnan, China	adjacent to type locality	GU981264	GU981112	GU981187	GU981445	He *et al*. [17]
*Chimarrogale himalayica*	Yunnan, China	adjacent to type locality	GU981263	/	/	/	He *et al*. [17]
*Chodsigoa hypsibia*	Beichuan, Sichuan, China	adjacent to type locality	KX765527	/	/	/	Chen *et al*. [27]
*Chodsigoa hypsibia*	Mengda, Qinghai, China		KX765528	/	/	/	Chen *et al*. [27]
*Chodsigoa hypsibia*	Mengda, Qinghai, China		KX765529	/	/	/	Chen *et al*. [27]
*Chodsigoa hypsibia*	Mengda, Qinghai, China		KX765530	/	/	/	Chen *et al*. [27]
*Chodsigoa hypsibia*	Mengda, Qinghai, China		KX765533	/	/	/	Chen *et al*. [27]
*Crocidura fuliginosa*	Yunnan, China	adjacent to type locality	GU981271	GU981117	GU981192	GU981450	He *et al*. [17]
*Crossogale hantu*	Selangor, Malaysia		MN149424	MN149427	/	MN149430	Abd Wahab *et al*. [28]
*Crossogale hantu*	Selangor, Malaysia		MN149423	MN149426	/	MN149429	Abd Wahab *et al*. [28]
*Crossogale hantu*	Selangor, Malaysia		MN149422	MN149428	/	MN149428	Abd Wahab *et al*. [28]
*Episoriculus caudatus*	Yunnan, China		GU981272	GU981118	GU981193	GU981451	He *et al*. [17]
*Episoriculus caudatus*	Yunnan, China		GU981273	/	/	/	He *et al*. [17]
*Episoriculus caudatus*	Yunnan, China		GU981274	GU981119	GU981194	GU981452	He *et al*. [17]
*Episoriculus caudatus*	Yunnan, China		GU981275	/	/	/	He *et al*. [17]
*Episoriculus caudatus*	Yunnan, China		GU981276	GU981120	GU981195	GU981453	He *et al*. [17]
*Episoriculus caudatus*	Yunnan, China		GU981277	/	/	/	He *et al*. [17]
*Episoriculus caudatus*	Phulchauki, Nepal		AB175114	/	/	/	Ohdachi *et al*. [29]
*Episoriculus caudatus*	Kurumsan, Nepal		AB175115	/	/	/	Ohdachi *et al*. [29]
*Episoriculus leucops*	Yunnan, China		GU981281	GU981122	GU981197	GU981455	He *et al*.[17]
*Episoriculus leucops*	Yunnan, China		GU981282	GU981123	GU981198	GU981456	He *et al*. [17]
*Episoriculus leucops*	Yunnan, China		GU981283	/	/	/	He *et al*. [17]
*Episoriculus leucops*	Yunnan, China		GU981284	/	/	/	He *et al*. [17]
*Episoriculus leucops*	Syng Gomba, Nepal		AB175111	/	/	/	Ohdachi *et al*. [29]
*Episoriculus macrurus*	Yunnan, China		GU981285	GU981122	GU981197	GU981455	He *et al*. [17]
*Episoriculus macrurus*	Yunnan, China		GU981286	GU981123	GU981198	GU981456	He *et al*. [17]
*Episoriculus macrurus*	Yunnan, China		GU981287	/	/	/	He *et al*. [17]
*Episoriculus macrurus*	Yunnan, China		GU981288	/	/	/	He *et al*. [17]
*Episoriculus macrurus*	Yunnan, China		GU981289	GU981124	GU981199	GU981457	He *et al*. [17]
*Episoriculus macrurus*	Yunnan, China		GU981290	GU981125	GU981200	GU981458	He *et al*. [17]
*Episoriculus macrurus*	Pokhara, Nepal		AB175109	/	/	/	Ohdachi *et al*. [29]
*Episoriculus macrurus*	Syabru, Nepal		AB175110	/	/	/	Ohdachi *et al*. [29]
*Episoriculus soluensis*	Gosainkund, Nepal	adjacent to type locality	AB175112	/	/	/	Ohdachi *et al*. [29]
*Episoriculus soluensis*	Gosainkund, Nepal	adjacent to type locality	AB175113	/	/	/	Ohdachi *et al*. [29]
*Nectogale elegans*	Yunnan, China		GU981294	/	/	/	He *et al*.[17]
*Nectogale elegans*	Yunnan, China		GU981293	GU981129	GU981204	GU981462	He *et al*. [17]
*Neomys fodiens*	Popova Sapka, North Macedonia		/	DQ630162	DQ630245	/	Dubey *et al*.[30]
*Neomys fodiens*	Hochsauerlandkreis, Germany	adjacent to type locality	/	GU981130	GU981205	GU981463	He *et al*.[17]
*Pantherina griselda*	Sichuan, China		KY249527	KY249532	MN199109	KY249544	Bannikova *et al*.[31]
*Pseudosoriculus fumidus*	Taiwan, China	adjacent to type locality	/	DQ630193	DQ630273	/	Dubey *et al*. [30]
*Pseudosoriculus fumidus*	Taiwan, China	adjacent to type locality	GU981278	GU981121	GU981196	GU981454	He *et al*.[17]
*Pseudosoriculus fumidus*	Taiwan, China	adjacent to type locality	GU981279	/	/	/	He *et al*.[17]
*Pseudosoriculus fumidus*	Taiwan, China	adjacent to type locality	GU981280	/	/	/	He *et al*. [17]
*Pseudosoriculus fumidus*	Taiwan, China	adjacent to type locality	AB175107	/	/	/	Ohdachi *et al*. [29]
*Pseudosoriculus fumidus*	Taiwan, China	adjacent to type locality	AB175108	/	/	/	Ohdachi *et al*. [29]
*Sorex bedfordiae*	Pingwu, Sichuan, China		OL585127	OL585986	OL585694	OL586703	Chen *et al*. [32]
*Sorex bedfordiae*	Pingwu, Sichuan, China		OL585125	OL585984	OL585692	OL586701	Chen *et al*. [32]
*Sorex bedfordiae*	Pingwu, Sichuan, China		OL585126	OL585985	OL585693	OL586702	Chen *et al*. [32]
*Sorex bedfordiae*	Pingwu, Sichuan, China		OL585128	OL585987	OL585695	OL586704	Chen *et al*. [32]
*Soriculus nigrescens*	Yunnan, China		GU981297	/	/	/	He *et al*. [17]
*Soriculus nigrescens*	Yunnan, China		GU981298	GU981132	GU981207	GU981465	He *et al*. [17]

Following He *et al*. [17] and Chen *et al*. [32, 33], we amplified the complete mitochondrial cytochrome b (*CYTB*) and three partial nuclear genes (apolipoprotein B (*APOB*), recombination-activating gene 2 (*RAG2*), and breast cancer 1 (*BRCA1*)). Primer sets are detailed in Table 4 [17, 30, 34, 35]. PCR amplifications were carried out in a 25 μl reaction volume mixture containing 12.5 μl of 2×Taq Master Mix (Vazyme, Nanjing, China), 1 μl of each primer, 1 μl of genomic DNA, and 9.5 μl of double-distilled water. PCR conditions for *CYTB* amplifications consisted of an initial denaturing step at 94°C for 5 min followed by 38 cycles of denaturation at 94°C for 45 s, annealing at 49°C for 45 s, an extension at 72°C for 90 s, and a final extension step at 72°C for 12 min. PCR conditions for nuclear genes were basically the same as those for *CYTB*, with a few modifications (annealing temperatures for each nuclear gene were *APOB* (49°C), *BRCA1* (51°C), and *RAG2* (52°C)). PCR products were checked on a 1.0% agarose gel and purified by ethanol precipitation. Purified PCR products were directly sequenced using the BigDye Terminator Cycle Kit v 3.1 (Applied Biosystems, Foster City, CA, USA) and an ABI 310 Analyzer (Applied Biosystems).

**Table 4:** Gene symbol, primer sequences and the best model of evolution for each gene segments used in the study.

Genes	Primers sequences (5’→3’)	The best model	Cited source
*CYTB*	GGACTTATGACATGAAAAATCATCGTTG	GTR+I+G	He *et al*. [17]
	GATTCCCCATTTCTGGTTTACAAGAC		He *et al*. [17]
*APOB*	GCAATCATTTGACTTAAGTG	HKY+G	Dubey *et al*. [30]
	GAGCAACAATATCTGATTGG		Dubey *et al*. [30]
*BRCA1*	TGAGAACAGCACTTTATTACTCAC	HKY+I+G	Dubey *et al*. [34]
	ATTCATGTTCCATATTGCTTATACTG		Dubey *et al*. [34]
*RAG2*	AGCTGCAGYCARTACCAYAARATGTA	HKY+I+G	Murphy *et al*. [35]
	AACTCAGCTGCATTKCCAATRTCACA		Murphy *et al*. [35]

To test phylogenetic relationships within the genus *Episoriculus*, sequences of these four genes from other Nectogalini species generated in previous studies [2, 17, 25–32] were downloaded from GenBank (Table 2).

### Sequence analyses

All *CYTB* sequences were aligned and examined. Screening for heterozygous nuclear gene fragments was performed in Mega 5 [36]. For analysis, we concatenated the three nuclear genes following He *et al*. [17]. Using all sequence data, phylogenetic analyses were conducted on: 1) *CYTB* data, 2) concatenated sequences for the three nuclear genes, and 3) each nuclear gene. Modeltest v 3.7 [37] was used to select the best-fitting evolutionary model, based on the Akaike Information Criterion in Table 3. MrBayes v 3.1.2 [38] was used for Bayesian analysis. *Crocidura fuliginosa* was selected as the outgroup. Each run was carried out with four Monte Carlo Markov chains (MCMCs), and 10,000,000 generations for single gene datasets and 30,000,000 generations for concatenated gene datasets. All runs were sampled every 10,000 generations. Convergences of runs were accepted when the average standard deviation of split frequencies was < 0.01. Ultrafast bootstrap values (UFBoot) of ≥ 95 and posterior probabilities (PP) of ≥ 0.95 were considered strong support [39].

### Species tree and species delimitation

To appraise current taxonomic systems based on morphology, and to evaluate trees derived from phylogenetic analyses, we used species delimitation and species tree construction based on coalescence theory [40, 41]. The basis of abductive theory-based species definition is to use the multispecies coalescent model to identify evolutionarily independent lineages in genomic data. which is significantly distinct from defining species as stable differences between monophyletic groups or taxa [42]. Since the *BEAST model requires that every gene segment in each sample be complete, we didn’t use all samples. We estimated the *BEAST coalescent species tree in Beast v 1.7.5 using partial nuclear and mitochondrial genes [43, 44]. Model settings were selected with reference to the optimal replacement model of each gene (Table 3). Each analysis was run for 100 ×10^6^ generations and sampled every 10,000 generations. Posterior distribution and effective sample size of each parameter were calculated using Tracer v 1.6. *BEAST analyses were repeated four times to ensure convergence on the same posterior distribution.

“Splits” (Species Limits by Threshold Statistics) v 1.0 14 was used for species delimitation in the context of R statistics. The program defines species using the generalized mixed Yule-coalescent model (GMYC) [45, 46]. Analysis requires a gene tree that has been corrected by a molecular clock as a reference; we use the bifurcated time tree constructed with Beast v 1.7.5 as basic data for this analysis.

BPP v 2.2 was used to species delimitation [47, 48]. Analyses of species boundaries were limited to *E*. *caudatus*, *E*. *sacratus*, and *E*. *umbrinus*; analyses used the combined nuclear gene data set. Since this program also requires a prior guide tree, and the topological structure of the guide tree will impact the result of species delimitation, we used the aforementioned species tree built by BEAST v 1.7.5 as the guide tree for BPP analysis [49]. For BPP, we set the Gamma prior distribution of the population size parameter (θs) to G (6, 6,000), and the initial differentiation time parameter (τ0) of the species tree to G (4, 1,000). Then 12 parameter combinations were generated using algorithms 0 and 1, and combining the values of Locusrate = 1 or Heredity = 1. The above 12 operations were performed on the two data groups; each operation was set to 1,000,000 generations of reverse-jump MCMC, and samples were taken every 10 generations; the first 10,000 generations were discarded [50].

Automatic barcode gap discovery (ABGD) software was used to divide samples based on genetic distance; samples within the same group were identified as one species [51]. *CYTB* sequences of the sample online submission to ABGD website (http://wwwabi.snv.Jussleu.fr/publicabgd/abgdweb.hml), the prior intraspecific divergence (P) ranged 0.001–0.1, and the minimum relative gap width (X) was 0.5.

We also used the Kimura 2-parameter (K2p) distance with 10,000 bootstrap replicates to summarize sequence divergences based on *CYTB* in MEGA5 [36].

### Morphometrics

Because we have found no evidence for sexual dimorphism in shrews in related taxa, we do not consider sex when selecting specimens and skulls [52]. All samples used for analysis were adults with intact skulls. Specimens of *Episoriculus* used for study have been deposited in the Sichuan Academy of Forestry, and Kunming Institute of Zoology. A total of 56 complete skulls of adult intact specimens were assigned to *E*. *macrurus* (17), *E*. *caudatus* (11), *E*. *umbrinus* (8), *E*. *leucops* (7), *E*. *sacratus* (6), and *E*. *soluensis* (3). Details of localities and museums are listed in S1 Table.

Measurements including head and body length (HBL), tail length (TL), hind foot length (HFL), and ear length (EL) were recorded from specimen labels or field notes. We measured the skulls of these specimens with a digital Vernier caliper (accuracy 0.01 mm). Eleven craniomandibular variables were taken: profile length (PL), cranial height (CH), cranial breadth (CB), interorbital breadth (IBO), palatoincisive length (PIL), postpalatal length (PPL), maxillary breadth (MB), upper toothrow length (UTR), maximum width across the upper second molars (M^2^–M^2^), mandibular length (ML), and lower toothrow length (LTR). Measurements dates were used for principal component analysis (PCA) in SPSS 22.0 (SPSS Inc., USA). Sample localities and measurements for each specimen are presented in S1 Table. Measuring methods followed Chen *et al*. [25], Yang *et al*. [53].

Cranial measurements were analyzed by PCA in SPSS v19.0 (SPSS Inc., USA). PCAs were conducted on log_10_ transformed variables on two data sets. Before analysis, the Kaiser–Meyer–Olkin (KMO) test (to check correlations or partial correlations between variables), and Bartlett’s sphere test (to determine if the correlation matrix is an identity matrix) were performed.

## Results

### Phylogenetic analysis

We obtained 69 mitochondrial sequences, and 156 nuclear sequences. All *CYTB*, *APOB*. *BRCA1* and *RAG2* files are available in GenBank (GenBank Accession No.: MK962157-MK962237, MN032125-MN032174, MN032175-MN032241, and MN032242-MN032310) (Table 2).

**Fig 2:**
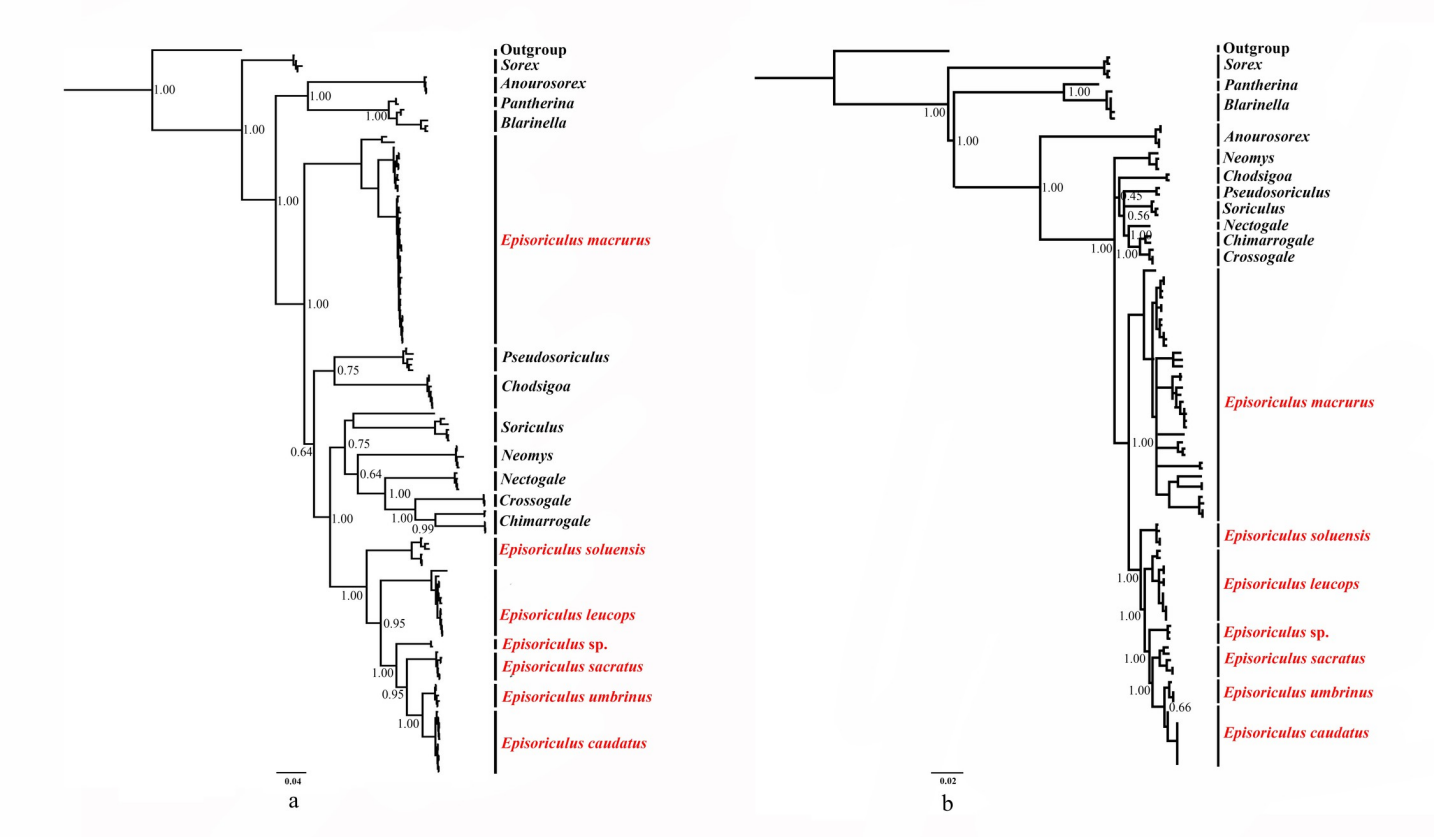
Bayesian phylogenetic analyses based on (a) *CYTB*, and (b) three concatenated nuclear genes. Numbers at nodes refer to Bayesian posterior probabilities. Scale bars represent substitutions per site.

Bayesian reconstruction using *CYTB* revealed eight monophyletic clades of *Episoriculus*, corresponding to *E*. *macrurus*, *E*. *soluensis*, *E*. *leucops*, *E*. *caudatus*, *E*. *umbrinus*, *E*. *sacratus*, and *E*. sp. (Fig 2A). *P*. *fumidus* clustered with *Chodsigoa* with strong support (PP = 0.95). *E*. *macrurus* represented a basal lineage of Nectogalini with strong support (PP = 1.00), and *E*. *soluensis*, *E*. *leucops*, *E*. *sacratus* and *E*. sp. formed a separate, strongly supported lineage (PP = 0.95–1.00). *E*. *umbrinus* and *E*. *caudatus*, located at the tip of this tree, formed sister clades, that were less-well supported (PP = 0.75).

Bayesian reconstruction using the three concatenated nuclear genes also revealed eight monophyletic clades (Fig 2B), but with a slightly different topological structure to the *CYTB* tree. *P*. *fumidus* mixed with *Chodsigoa* and *Soriculus*, with weak support. All species of *Episoriculus* formed a monophyletic clade, with *E*. *macrurus* at its base. Lineages of *E*. *soluensis*, *E*. *leucops*, *E*. *sacratus*, and *E*. sp. were strongly supported (PP = 1.00). *E*. *umbrinus* and *E*. *caudatus* formed sister clades at the tree’s tip, although support for them was weak (PP = 0.66).

**Fig 3:**
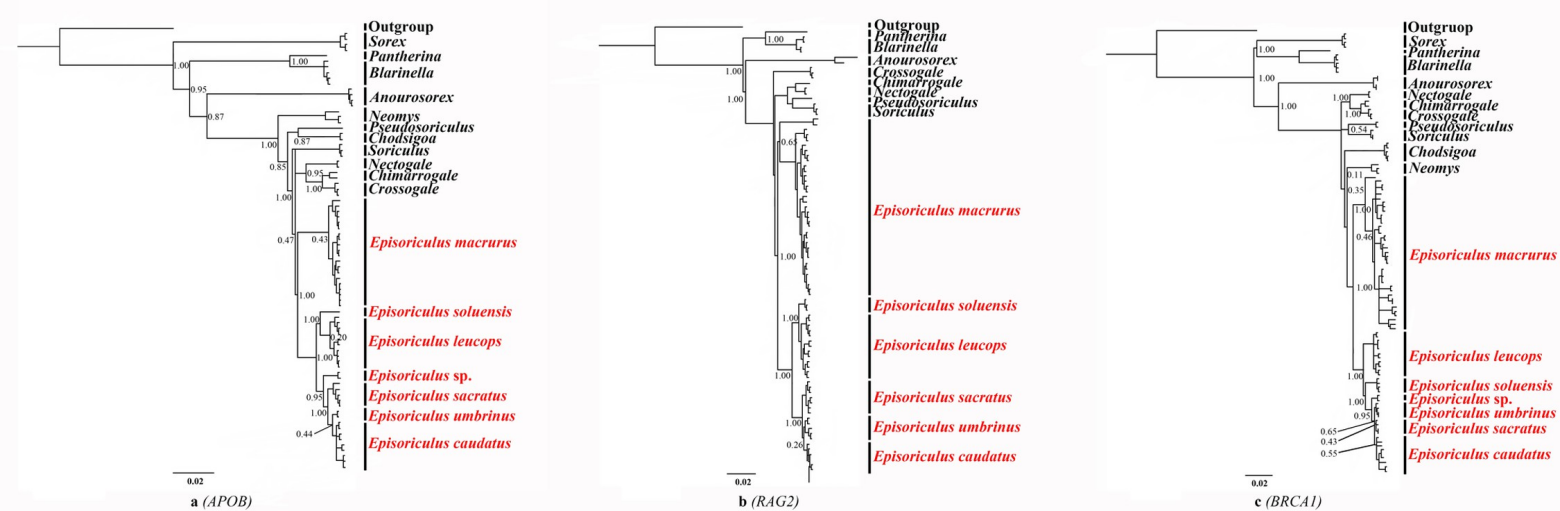
Bayesian phylogenetic analyses based on (a) *APOB*, (b) *RAG2*, and (c) *BRCA1* genes. Numbers at nodes refer to Bayesian posterior probabilities. Scale bars represent substitutions per site.

Structures of three individual nuclear gene trees differed from the *CYTB* and three concatenated nuclear gene trees (Fig 3), with some nodes having very low support. *P*. *fumidus* did not cluster with *Episoriculus*. *E*. *macrurus*, *E*. *soluensis*, *E*. *leucops*, *E*. sp., and *E*. *sacratus* remained monophyletic with strong support based on *APOB* and *RAG2* (PP = 1.00) genes, and *E*. *umbrinus* and *E*. *caudatus* formed a sister group with weak support. *E*. *sacratus*, *E*. *umbrinus* and *E*. *caudatus* were mixed on the tree based on the *BRCA1* gene, with very low support.

### Species delimitation

**Fig 4:**
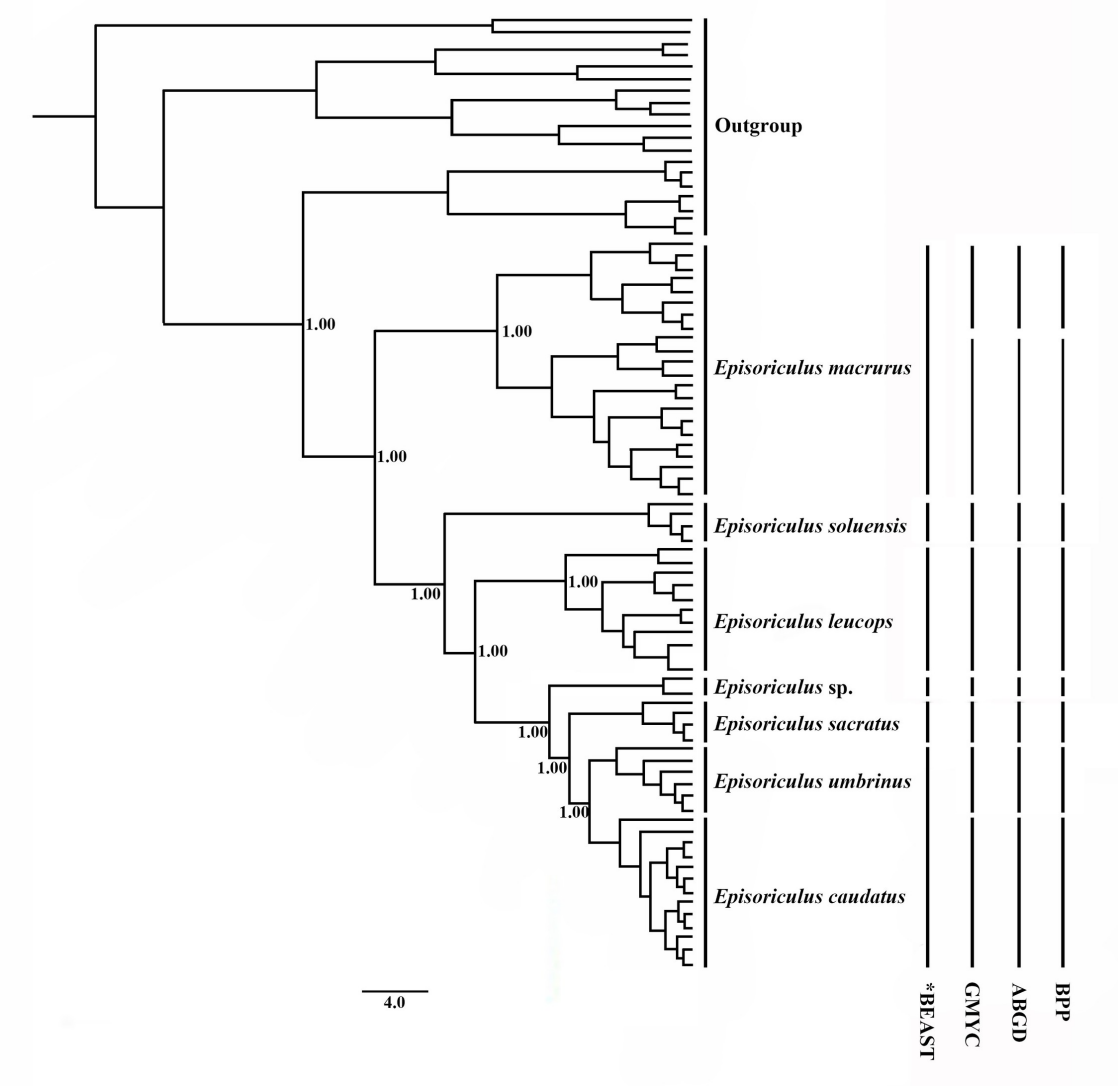
Results of species delimitation using splits, GMYC, BPP, and species trees reconstructed using the *BEAST model. Node numbers indicate Bayesian posterior probabilities supporting each clade as two putative species.

The topology of *BEAST species’ trees differed slightly from those of mitochondrial and nuclear genes (Fig 4). *E*. *macrurus*, *E*. *soluensis*, *E*. *leucops*, *E*. *sacratus*, and *E*. sp. also had high support in these trees (PP = 1.00). *E*. *umbrinus* and *E*. *caudatus* were sister clades, but with weak support in the tree (PP = 0.75). This result did not support recognizing *E*. *umbrinus* as a distinct species.

GMYC analysis reveals five clades as valid species (Fig 4), of which *E*. *soluensis*, *E*. *leucops*, and *E*. sp. are separate, and *E*. *macrurus* comprised two species, with individuals from Sichuan and Yunnan provinces differing. This analysis suggests that *E*. *caudatus*, *E*. *sacratus* and *E*. *umbrinus* are conspecific.

For the ABGD analysis, the transition/transversion value (3.5) first calculated by Mega 5 was used as the starting parameter, with 0.5, 1.0, 1.5 and 2.0 used as relative gap widths. The 81 samples divided into eight species: *E*. *caudatus*, *E*. *macrurus*, *E*. *sacratus*, *E*. *soluensis*, *E*. *leucops*, *E*. *macrurus* (Sichuan samples), *E*. *macrurus* (Yunnan samples), and *E*. sp. (Fig 4 and S2 Table).

BPP analysis revealed *E*. *caudatus* and *E*. *umbrinus* to be separate species in 12 groups of BPP data based on the combined nuclear gene data set, with support for *E*. *sacratus* being a valid taxon also being high (Fig 4 and S3 Table).

Kimura-2-parameter (K2p) distances between *Episoriculus* species ranged 0.027–0.160 (Table 5). The average K2p distance between *P*. *fumidus* and *Episoriculus* species was 0.177. The K2p distance between *E*. *caudatus* and *E*. *sacratus* was 0.067, and between *E*. *caudatus* and *E*. *umbrinus*, 0.027, and *E*. *sacratus* and *E*. *umbrinus*, 0.071.

**Table 5:** The Kimura-2-parameter distances between *Episoriculus* species based on the *CYTB* gene.

No.	Species	1	2	3	4	5	6	7
1	*Episoriculus caudatus*							
2	*Episoriculus sacratus*	0.066						
3	*Episoriculus umbrinus*	0.027	0.071					
4	*Episoriculus* sp.	0.083	0.088	0.099				
5	*Episoriculus soluensis*	0.113	0.121	0.128	0.129			
6	*Episoriculus leucops*	0.112	0.133	0.131	0.126	0.139		
7	*Episoriculus macrurus*	0.149	0.156	0.160	0.148	0.159	0.154	
8	*Pseudosoriculus fumidus*	0.181	0.184	0.192	0.177	0.163	0.189	0.154

### Morphology

Morphological data (HBL, TL, HFL, EL, and body weight (BW)) of 56 specimens with intact skulls are presented in Table 6. The TL of *E*. *macrurus* ranged 71–106mm, while values for congeners ranged 46–82.5 mm. The HFL of *E*. *macrurus* was 15–16 mm, while for other species it was 12–13 mm. *E*. *macrurus* had the lowest HBL/TL ratio (0.64), and its TL was ~1.5 times its HBL, while the HBL of congeners was approximately equal to or greater than TL. *E*. *leucops* had the largest body, and *E*. *sacratus* differed from *E*. *caudatus* and *E*. *umbrinus* in HBL/TL values. Morphological indices are detailed in Table 7, and complete measurement data are provided in S1 Table.

**Table 6:** The results of body morphologic measurements.

Species	N	BW	HBL	TL	HFL	HBL / TL
*E*. *caudatus*	14	5.000±0.327	56.750±1.858	54.750±0.491	12.125±0.125	1.04
*E*. *sacratus*	6	5.666±0.211	57.134±0.632	62.166±2.249	13.500±0.671	0.92
*E*. *umbrinus*	9	5.000±0.239	61.333±0.816	60.222±0.547	13.000±0.231	1.01
*E*. *leucops*	7	7.714±0.286	62.714±0.056	60.143±1.534	13.143±0.143	1.04
*E*. *macrurus*	17	5.000±0.413	58.158±1.732	91.333±1.201	15.667±0.333	0.64
*E*. *soluensis*	4	6.000 ±0.210	64.333±0.666	61.333±0.088	12.000±0.531	1.04

**Table 7:** Morphological measurement data of *Episoriculus* species skulls.

Species	N	CIL	IOB	CB	BH	MB	PIL	PPL	UTR	M^2^-M^2^	ML	LTR
*E*. *caudatus*	14	18.327 ±0.057	3.311 ±0.044	8.769 ±0.374	5.907 ±0.068	1.391 ±0.018	8.104 ±0.059	8.067 ±0.039	8.1464 ±0.058	4.525 ±0.038	11.258 ±0.056	7.2657 ±0.058
*E*. *sacratus*	6	18.206 ±0593	3.430 ±0.052	8.578 ±0.082	6.030 ±0.100	1.381 ±0.237	7.840 ±0.056	8.035 ±0.103	7.773 ±0.073	4.565 ±0.029	11.036 ±0.090	6.981 ±0.046
*E*. *umbrinus*	8	18.286 ±0.070	3.523 ±0.054	8.513 ±0.045	5.628 ±0.045	1.371 ±0.024	8.208 ±0.063	8.126 ±0.088	8.052 ±0.046	4.457 ±0.049	11.1789 ±0.077	7.3933 ±0.0763
*E*. *leucops*	7	19.307 ±0.111	3.708 ±0.377	9.068 ±0.448	5.858 ±0.046	1.391 ±0.341	8.658 ±0.036	8.876 ±0.797	8.416 ±0.082	4.564 ±0.062	11.693 ±0.130	7.354 ±0.147
*E*. *macrurus*	18	17.577 ±0.060	3.309 ±0.191	8.456 ±0.057	5.761 ±0.050	1.290 ±0.012	7.633 ±0.051	7.813 ±0.079	7.565 ±0.056	4.184 ±0.045	10.618 ±0.051	6.781 ±0.036
*E*. *soluensis*	4	17.896 ±0.003	3.603 ±0.052	9.327 ±0.097	5.763 ±0.233	1.706 ±0.328	8.067 ±0.296	7.976 ±0.024	7.767 ±0.084	4.873 ±0.003	12.320 ±0.055	7.497 ±0.084

Bartlett’s test rejected the null hypothesis (χ^2^ = 568.01, P = 0.000), indicating that the data were spherical and variables were somewhat independent of each other. A KMO of 0.813 indicated a strong correlation existed among the various skull data, which was suitable for factor analysis. Two principal components explaining 74.69% of morphological variation were extracted from the analysis. Factor loading values were most positive, indicating that it was mainly related to overall skull size (Table 8). Features with factor loads > 0.8 included PIL, ML, CIL, UTR, and LTR.

**Table 8:** Character loadings, eigenvalues, and proportion of variance explained by the first two axes (PC 1 and PC 2) of a principal component analysis using the log10-transformed measurements of *Episoriculus*. The meanings of variable abbreviations are given in the Materials and Methods Section.

Measurement	Principal component (PC)	
	1	2
PIL	0.888	-0.340
ML	0.886	0.216
CIL	0.876	-0.247
UTR	0.841	-0.279
LTR	0.804	-0.003
CB	0.750	0.290
M^2^-M^2^	0.733	0.502
PPL	0.704	-0.409
IOB	0.682	-0.258
MB	0.592	0.722
CH	0.030	0.537
Variance explained	61.022	13.664

**Fig 5:**
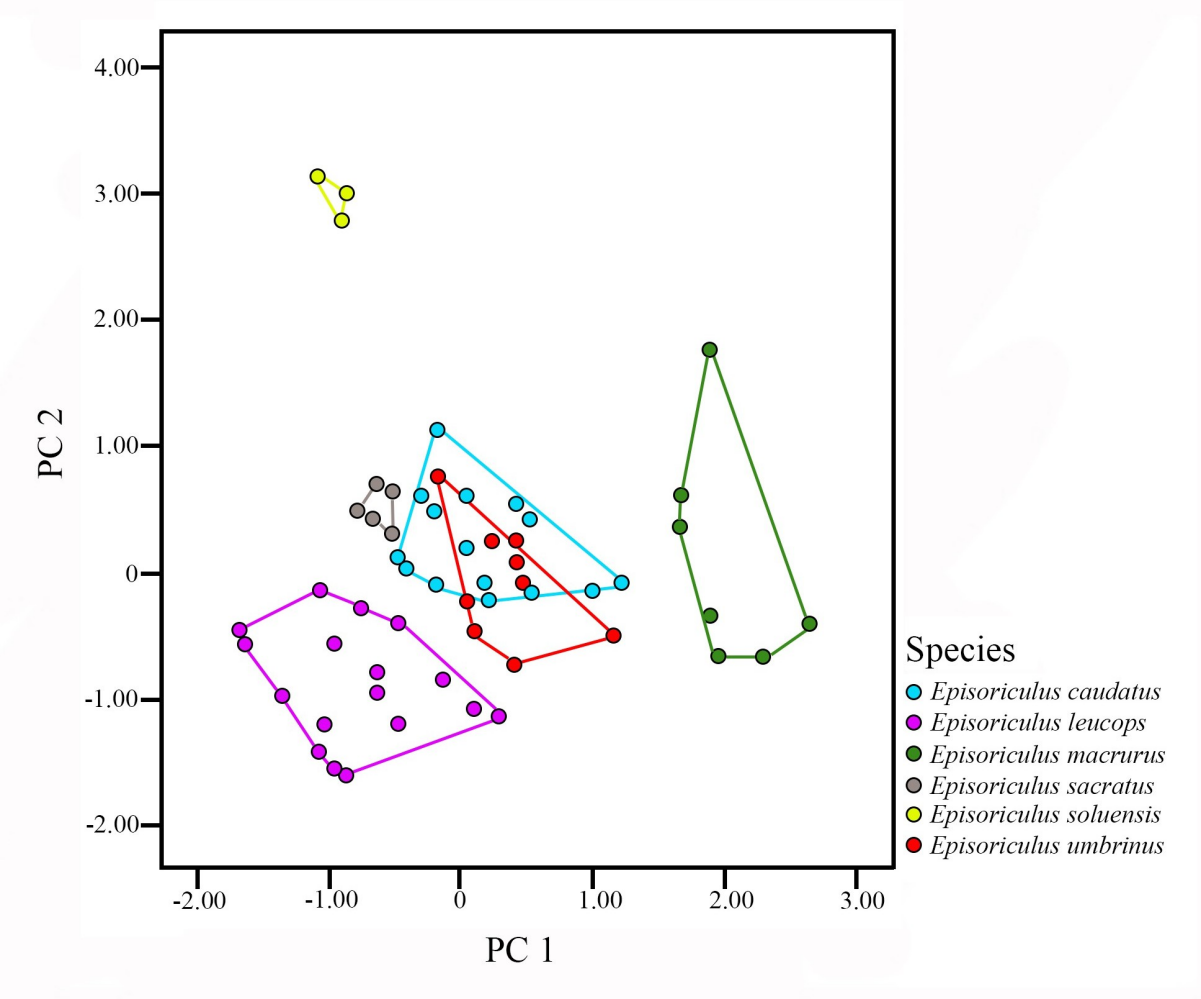
Results of principal component analysis of *Episoriculus* taxa based on 19 log_10_ transformed craniodental measurements.

Using PC 1 and PC 2 maps (Fig 5), *E*. *macrurus* plotted in the positive region of PC 2, while other species were mainly in the negative region of PC 2. The larger *E*. *leucops* plotted in the negative region of PC 2, and *E*. *soluensis* plotted in the positive region of PC 2. *E*. *sacratus* was distinguished with *E*. *umbrinus* and *E*. *caudatus*, with the latter two species being mixed and not effectively differentiated.

**Fig 6:**
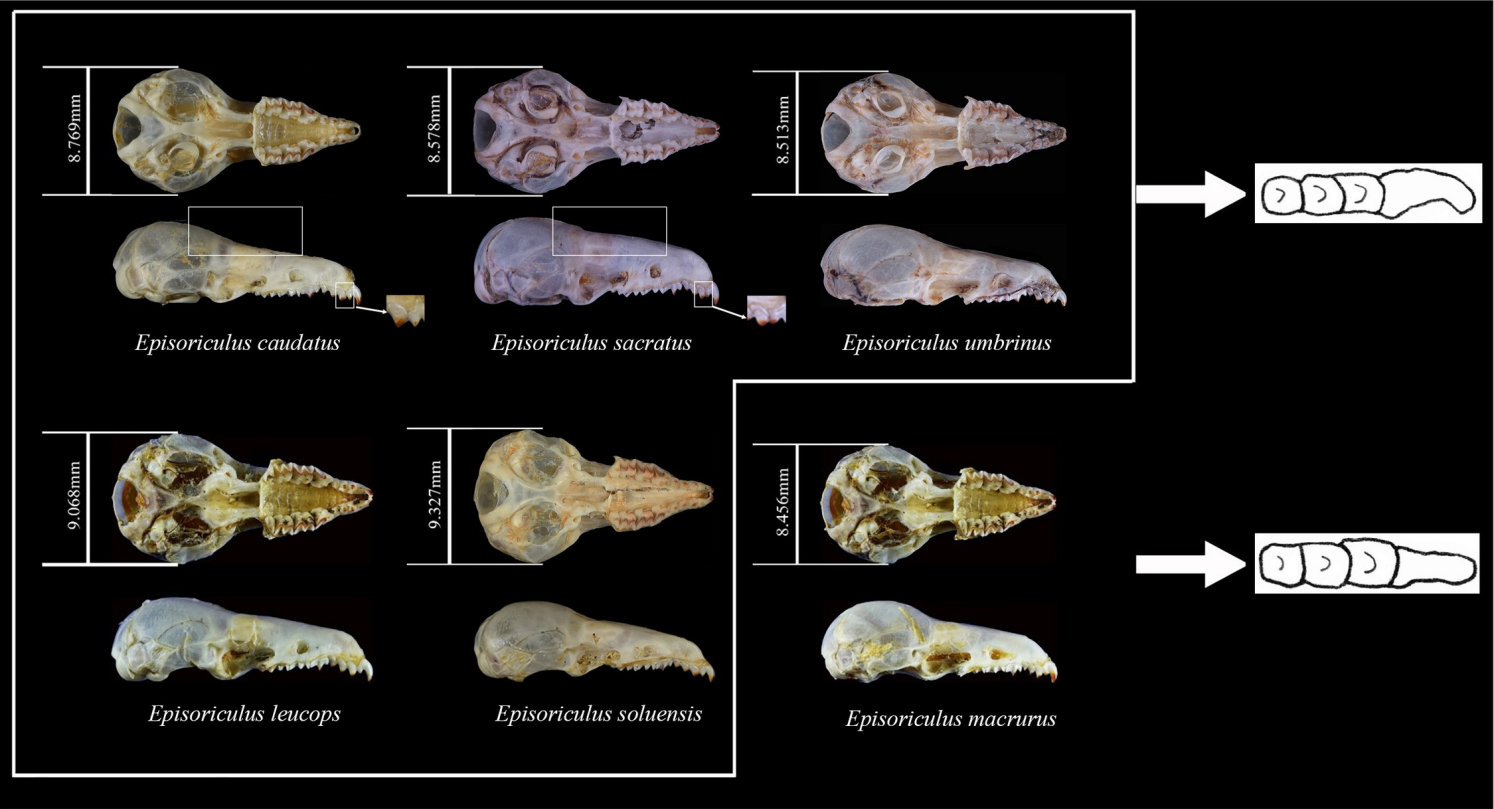
Comparison of *Episoriculus* skulls.

Compared to skulls of other *Episoriculus* species (Fig 6), the braincase of *E*. *macrurus* is more dome-shaped, the rostrum is shorter; and the upper unicuspids are quadrangular and wider than long (those of other species are similarly sized). Compared to skulls of *E*. *caudatus*, the frontal region of the skull of *E*. *sacratus* is more arched, and the posterior cusp of its upper incisor is lower than its first unicuspid, whereas the height of the posterior cusp of the upper incisor and first unicuspid of *E*. *caudatus* are similar.

## Discussion

In the study, we collected a huge number of shrews in China and, via morphological and molecular analysis, concluded that genus *Episoriculus* contains six distinct species and one cryptic species. The majority of *Episoriculus* species have their type origin in India. We further validated the accuracy of our species classification by utilizing sequence collected in India from Ohdachi *et al*. [29]. We did comprehensive studies on some species whose classification is contested.

Thomas [54] described six specimens from Mount Emei as *E*. *sacratus*, and considered it most likely the local representative of *E*. *caudatus*, from which they differed in having a much smaller braincase. Allen [55] described the subspecies *E*. *caudatus umbrinus* from Mucheng, Yunnan, which was most alike *E*. *sacratus*, but differed from it in its much darker-brown color and in having a uniformly dark rather than bicolor tail. Allen [14] demoted *E*. *sacratus* to a subspecies of *E*. *caudatus*—an opinion with which Ellerman and Morrison-Scott [1] agreed. Hoffmann [7] examined many specimens and concluded that there were similarities in skull and cranial size between *E*. *c*. *caudatus*, *E*. *c*. *sacratus* and *E*. *c*. *umbrinus*. Wilson and Reeder [9, 13] recognized these three taxa to be distinct species based on karyotypes and differences in skull size, while Motokawa and Lin [15] considered *E*. *sacratus* and *E*. *umbrinus* to be subspecies of *E*. *caudatus*. Motowaka *et al*. [16] considered the larger *E*. *caudatus* and smaller *E*. *sacratus* to be distinct species, and included three subspecies: *E*. *soluensis*, *E*. *umbrinus*, and *E*. *sacratus* as subspecies of *E*. *caudatus*. Wilson and Mittermeier [3] elevated *E*. *umbrinus* to full species without reason, while Wei *et al*. [56] regarded it to be a subspecies of *E*. *caudatus*. Our data support recognizing *E*. *sacratus* as a valid species, and recognizing *E*. *umbrinus* to be a subspecies of *E*. *caudatus*. In morphology, *E*. *sacratus* can be differentiated from *E*. *caudatus*, but *E*. *umbrinus* cannot. Our data do not support the opinion of Motowaka *et al*. [16]. For the two species, we considered that the Jinsha River and Hengduan Mountains shut off communication between *E sacratus* and *E*. *caudatus*, limiting the species to the edge of the western Sichuan Plateau. And *E*. *caudatus* is widely distributed in northeastern India, northern Burma, northern Vietnam, and Xizang, Yunnan, Guizhou, China [2, 3, 17, 57].

Gruber [23] described *E*. *soluensis* and considered it a separate species. Abe [58] reviewed specimens collected in central Nepal and compared them with those from eastern Nepal by Gruber [23], and both suggested that *E*. *soluensis* was a subspecies of *E*. *caudatus*, but also possibly synonymous with *E*. *sacratus*. Hoffmann [7] treated *E*. *soluensis* as a synonym of *E*. *caudatus*—an opinion with which Wilson and Reeder [9, 13] and Motokawa and Lin [15] agreed. Ohdachi *et al*. [29] regarded two samples from Nepal to be *E*. *caudatus soluensis* following Abe [58], and sequenced *CYTB*. Abramov *et al*. [2] then used these two sequences to reconstruct a system tree, and after determining that *E*. *soluensis* constituted a distinct clade from *E*. *caudatus*, advocated for them being treated as distinct species. In our tree, four samples from Yadong and Nyalam cluster with the two *E*. *soluensis* samples of Ohdachi *et al*. [29], and these four specimens are similar in having dark-brown ventral hair, and light-yellowish-brown dorsal hair. The tail length of our four specimens is longer than the head length, the skull parietal bone is relatively protruding, there are four upper single cusp teeth, the posterior cusp teeth of the maxillary incisor are similar in height to the first upper single cusp teeth, and the cusp teeth are light brown. These features are basically consistent with Gruber’s [23] original description, and the description of *E*. *soluensis* of Wilson and Mittermeier [3]. Accordingly, we regard *E*. *soluensis* to be a distinct species, and report it for the first time from China. And We discovered that this species lives on both sides of the middle Himalayas (Nepal, northeast India, and Shigatse region, China).

While Ellerman and Morrison-Scott [1] regarded *Episoriculus baileyi* to be a subspecies of *E*. *caudatus*, Abe [58, 59] identified the two to be morphologically distinct and sympatric in Nepal. Hoffmann [7] examined species from Burma and Nepal and considered *E*. *baileyi* to be a subspecies of *E*. *leucops*, an opinion with which Wilson and Reeder [9, 13] agreed. Based on external and cranial morphology, Motokawa and Lin [15] re-evaluated the taxonomic status of *E*. *baileyi*, and considered it to be a valid species of *Episoriculus*. This species could be distinguished from other *Episoriculus* in the combination of its robust first upper incisor, long rostrum and upper unicuspid row, large tympanic ring, and high ascending ramus of the mandible—an opinion with which Wilson and Mittermeier [3] agreed. Because of a lack of specimens, we cannot investigate the status of *E*. *baileyi*. We provisionally follow Motokawa and Lin [15], but the taxonomic status of this species requires further investigation.

While Ellerman and Morrison-Scott [1] considered *E*. *fumidus* to be a subspecies of *E*. *caudatus*, Jameson and Jones [11] considered it to be a distinct species based on its geographical isolation and morphological divergence—an opinion with which Hoffmann [7], Wilson and Reeder [9, 13], and Motokawa and Lin [15] agreed. Dubey *et al*. [29] inferred that *E*. *fumidus* (the only representative of the genus in their study) was a sister group of *Chodsigoa* with strong support in the *APOB* gene tree—an opinion with which He *et al*. [17] agreed. Based on the sequence of Dubey *et al*. [29] and He *et al*. [17], Abramov *et al*. [2] regarded *fumidus* do not belong in *Episoriculus*, and established the genus *Pseudosoriculus* for it—an opinion supported by our analyses.

We identify what appears to be a new cryptic species (*E*. sp.) from low-elevation areas in Motuo County, Xizang, which forms a separate branch in our system tree. However, with only two specimens available, we cannot accurately describe its morphology. Further specimens and molecular data are required to accurately resolve the taxonomic status of this taxon.

Our phylogenic analysis consistently roots *E*. *macrurus* as an individual lineage. This species, which has large genetic distance from congeners, in phylogenetic trees is usually located in the outermost or most basal part of the genus *Episoriculus*. It has the longest tail in the genus, and differs from congeners in skull and tooth morphology. For these reasons we speculate it retains some of the most primitive traits in genus *Episoriculus* or Nectogalini. However, in phylogenetic trees based on different genes, the position of a varies: in the phylogenetic tree based on mitochondrial gene, *E*. *macrurus* is at the base of tribe Nectogalini and forms a single monophyletic group, whereas in the nuclear gene-based tree, *E*. *macrurus* is included in Nectogalini and clustered with other species of *Episoriculus* on the same clade. Similar conflicting phylogenetic signals have been reported in other studies [60]. This phenomenon could be explained from the perspectives of genetic background [61], ancient hybridization [62], incomplete lineage sorting [63], adaptive evolution, and burst Formula speciation [64]. The long tail and developed hind feet of this species lend it a semi-arboreal appearance. This specific niche adaption, which is not shared by other species, may result in huge differences in energy metabolism patterns between *E*. *macrurus* and other species of Nectogalini. This was reflected in mitochondria, which caused early differentiation in the species tree built using mitochondrial genes, resulting in incomplete lineage sorting. While neither mtDNA nor nDNA alone resolved phylogenetic relationships in the genus *Episoriculus*, combining data from these two genetic pathways did improve results. Species tree construction in the coalescent framework also produced a consistent topology with high statistical support. Therefore, we deem that a combined approach using mitochondrial and nuclear gene information is more appropriate for resolving phylogenetic relationships in the genus *Episoriculus*.

## Conclusion

Based on molecular and morphological analyses, the genus *Episoriculus* comprises at least six valid species: *E*. *baileyi*, *E*. *caudatus*, *E*. *leucops*, *E*. *macrurus*, *E*. *sacratus*, *E*. *soluensis*, and the potentially undescribed *E*. sp.

## Supporting information

S1 Table External and selected cranial measurements of *Episoriculus* species. (DOCX)

S2 Table Results of ABGD species definition based on *CYTB* gene. (DOCX)

S3 Table Posterior probabilities supporting three species (*Episoriculus caudatus*, *E*. *sacratus*, and *E*. *umbrinus*) as potential species using different algorithms and priors. (DOCX)
