# Supplementary material for: Integrative phylogenetic analysis of the genus Episoriculus (Mammalia: Eulipotyphla: Soricidae)
Source: PLoS One. 2025 Jan 17;20(1):e0299624. doi: 10.1371/journal.pone.0299624 (PMC11981537; doi:10.1371/journal.pone.0299624)
Supplement: S1 Table — (DOCX) [file pone.0299624.s001.docx]

**S1 Table External and selected cranial measurements of *Episoriculus* species**

| Number | Sample ID | Museum | Species | CIL | IOB | CB | BH | MB | PIL | PPL | UTR | M^2^-M^2^ | ML | LTR |
| --- | --- | --- | --- | --- | --- | --- | --- | --- | --- | --- | --- | --- | --- | --- |
| 1 | XZRAP07010 | SAF | *Episoriculus caudatus* | 18.04 | 3.1 | 8.81 | 5.52 | 1.41 | 7.89 | 8.32 | 7.99 | 4.45 | 11.02 | 6.92 |
| 2 | XZRAP08003 | SAF | *Episoriculus caudatus* | 18.19 | 3.11 | 8.73 | 6.42 | 1.45 | 8.1 | 8.02 | 8.12 | 4.43 | 11.07 | 7.03 |
| 3 | XZLZT02001 | SAF | *Episoriculus caudatus* | 18.16 | 3.15 | 8.74 | 6.1 | 1.46 | 8.05 | 7.94 | 8.11 | 4.47 | 11.19 | 7.25 |
| 4 | CYMD0103 | SAF | *Episoriculus caudatus* | 18.28 | 3.23 | 8.89 | 5.89 | 1.42 | 8.31 | 8.17 | 8.39 | 4.74 | 11.23 | 7.32 |
| 5 | XZLZT03002 | SAF | *Episoriculus caudatus* | 18.26 | 3.24 | 8.87 | 5.67 | 1.48 | 8.06 | 7.89 | 8.41 | 4.68 | 11.41 | 7.68 |
| 6 | XZLZT03001 | SAF | *Episoriculus caudatus* | 18.23 | 3.29 | 8.8 | 5.97 | 1.45 | 7.84 | 8.31 | 8.1 | 4.69 | 11.17 | 7.28 |
| 7 | CY01 | SAF | *Episoriculus caudatus* | 18.35 | 3.35 | 8.45 | 5.88 | 1.35 | 7.75 | 7.85 | 7.69 | 4.58 | 11.01 | 7.22 |
| 8 | XZRAP05005 | SAF | *Episoriculus caudatus* | 18.49 | 3.4 | 8.85 | 6.19 | 1.43 | 8.37 | 8.1 | 8.16 | 4.74 | 11.13 | 6.98 |
| 9 | ELSDGA02004 | SAF | *Episoriculus caudatus* | 18.13 | 3.49 | 8.49 | 6.28 | 1.43 | 7.69 | 7.88 | 7.73 | 4.59 | 10.65 | 6.88 |
| 10 | XZRAP05004 | SAF | *Episoriculus caudatus* | 18.18 | 3.3 | 8.51 | 5.85 | 1.36 | 8.04 | 8.15 | 8.11 | 4.39 | 11.48 | 7.34 |
| 11 | XZRAP06002 | SAF | *Episoriculus caudatus* | 18.51 | 3.54 | 8.7 | 5.79 | 1.31 | 8.12 | 8.06 | 8.16 | 4.38 | 11.44 | 7.38 |
| 12 | CY17 | SAF | *Episoriculus caudatus* | 18.75 | 3.64 | 8.91 | 5.63 | 1.29 | 8.54 | 8.1 | 8.42 | 4.44 | 11.59 | 7.57 |
| 13 | XZRAP05007 | SAF | *Episoriculus caudatus* | 18.73 | 3.44 | 8.89 | 5.82 | 1.3 | 8.34 | 8.15 | 8.35 | 4.39 | 11.62 | 7.42 |
| 14 | CY43 | SAF | *Episoriculus caudatus* | 18.22 | 3.14 | 8.87 | 5.71 | 1.31 | 8.11 | 7.98 | 8.25 | 4.36 | 11.2 | 7.28 |
| 15 | XZRAP07001 | SAF | *Episoriculus caudatus* | 18.19 | 3.43 | 8.75 | 6.27 | 1.46 | 7.94 | 7.91 | 7.79 | 4.61 | 11.06 | 7.05 |
| 16 | NJ13024 | SAF | *Episoriculus caudatus* | 19.83 | 3.86 | 9.2 | 5.71 | 1.55 | 8.64 | 8.48 | 8.81 | 4.76 | 12.42 | 8.12 |
| 17 | NJ13138 | SAF | *Episoriculus caudatus* | 19.26 | 3.58 | 9.18 | 5.99 | 1.44 | 8.6 | 8.66 | 8.28 | 4.44 | 11.6 | 7.6 |
| Number | Sample ID | Museum | Species | CIL | IOB | CB | BH | MB | PIL | PPL | UTR | M^2^-M^2^ | ML | LTR |
| 18 | 811113 | KIZ | *Episoriculus leucops* | 19.03 | 3.61 | 8.89 | 5.95 | 1.33 | 8.77 | 8.65 | 8.27 | 4.44 | 11.54 | 7.33 |
| 19 | 811137 | KIZ | *Episoriculus leucops* | 19.01 | 3.65 | 9.01 | 5.67 | 1.31 | 8.61 | 8.91 | 8.22 | 4.45 | 11.41 | 7.11 |
| 20 | 811138 | KIZ | *Episoriculus leucops* | 19.44 | 3.78 | 9.01 | 5.89 | 1.33 | 8.81 | 9.01 | 8.57 | 4.61 | 11.72 | 7.21 |
| 21 | 811142 | KIZ | *Episoriculus leucops* | 19.46 | 3.74 | 9.18 | 5.89 | 1.45 | 8.63 | 8.59 | 8.27 | 4.81 | 11.73 | 7.06 |
| 22 | 811147 | KIZ | *Episoriculus leucops* | 19.12 | 3.74 | 9.01 | 5.91 | 1.33 | 8.55 | 8.99 | 8.49 | 4.44 | 11.43 | 7.05 |
| 23 | WLCJT070720 | SAF | *Episoriculus macrurus* | 18.1 | 3.31 | 8.66 | 5.82 | 1.35 | 7.92 | 8.06 | 7.69 | 4.12 | 10.64 | 6.88 |
| 24 | WL06-06-D2-03 | SAF | *Episoriculus macrurus* | 17.3 | 3.32 | 8.42 | 5.88 | 1.29 | 7.52 | 7.5 | 7.23 | 4.09 | 10.32 | 6.62 |
| 25 | 06-8-31-2-2 | SAF | *Episoriculus macrurus* | 18.08 | 3.24 | 8.73 | 6.01 | 1.33 | 8.01 | 7.96 | 7.98 | 4.17 | 10.36 | 6.65 |
| 26 | ELSLCA03001 | SAF | *Episoriculus macrurus* | 17.74 | 3.15 | 8.57 | 6.17 | 1.35 | 7.54 | 7.59 | 7.64 | 4.52 | 10.52 | 6.77 |
| 27 | JJSA506 | SAF | *Episoriculus macrurus* | 17.67 | 3.3 | 8.24 | 5.85 | 1.3 | 7.91 | 8.74 | 7.76 | 4.41 | 10.81 | 6.9 |
| 28 | JJSA472 | SAF | *Episoriculus macrurus* | 17.42 | 3.3 | 8.21 | 5.94 | 1.32 | 7.25 | 7.43 | 7.34 | 4.36 | 10.41 | 6.62 |
| 29 | ELSBTDBS01013 | SAF | *Episoriculus macrurus* | 17.55 | 3.27 | 8.22 | 6.03 | 1.32 | 7.6 | 7.69 | 7.67 | 4.37 | 10.74 | 6.76 |
| 30 | MGDW0502 | SAF | *Episoriculus macrurus* | 17.42 | 3.29 | 8.13 | 5.78 | 1.29 | 7.3 | 7.4 | 7.26 | 4.16 | 10.91 | 7 |
| 31 | NJ13090 | SAF | *Episoriculus macrurus* | 17.37 | 3.39 | 8.58 | 5.74 | 1.22 | 7.43 | 7.6 | 7.28 | 3.86 | 10.39 | 6.63 |
| 32 | SM15017 | SAF | *Episoriculus macrurus* | 17.35 | 3.27 | 8.19 | 5.77 | 1.21 | 7.47 | 7.87 | 7.41 | 4.1 | 10.44 | 6.58 |
| 33 | WL14087 | SAF | *Episoriculus macrurus* | 17.88 | 3.44 | 8.66 | 5.73 | 1.36 | 7.73 | 7.94 | 7.68 | 4.15 | 11.01 | 6.89 |
| 34 | NJ13032 | SAF | *Episoriculus macrurus* | 17.44 | 3.43 | 8.37 | 5.58 | 1.29 | 7.5 | 7.89 | 7.22 | 4.06 | 10.44 | 6.63 |
| 35 | NJ13123 | SAF | *Episoriculus macrurus* | 17.34 | 3.24 | 8.29 | 5.48 | 1.21 | 7.74 | 7.44 | 7.73 | 3.82 | 10.75 | 6.73 |
| Number | Sample ID | Museum | Species | CIL | IOB | CB | BH | MB | PIL | PPL | UTR | M^2^-M^2^ | ML | LTR |
| 36 | NJ13124 | SAF | *Episoriculus macrurus* | 17.46 | 3.4 | 8.55 | 5.63 | 1.29 | 7.71 | 7.91 | 7.64 | 4.14 | 10.71 | 7.02 |
| 37 | NJ13125 | SAF | *Episoriculus macrurus* | 17.52 | 3.39 | 8.42 | 5.48 | 1.24 | 7.74 | 8.04 | 7.62 | 4.17 | 10.58 | 7.02 |
| 38 | YN15171 | SAF | *Episoriculus macrurus* | 17.49 | 3.22 | 8.5 | 5.54 | 1.28 | 7.73 | 7.89 | 7.84 | 4.26 | 10.57 | 6.78 |
| 39 | JJSA531 | SAF | *Episoriculus macrurus* | 17.68 | 3.29 | 9.01 | 5.51 | 1.28 | 7.66 | 7.87 | 7.61 | 4.37 | 10.91 | 6.79 |
| 40 | ELSMYPA02005 | SAF | *Episoriculus sacratus* | 18.28 | 3.33 | 8.8 | 5.74 | 1.45 | 7.84 | 7.81 | 7.69 | 4.62 | 11.01 | 6.93 |
| 41 | ELSLCC03002 | SAF | *Episoriculus sacratus* | 18.16 | 3.31 | 8.63 | 5.97 | 1.43 | 7.81 | 7.79 | 7.67 | 4.64 | 11.11 | 7.01 |
| 42 | ELSLCA03005 | SAF | *Episoriculus sacratus* | 18.09 | 3.4 | 8.96 | 6.24 | 1.43 | 7.82 | 7.83 | 7.65 | 4.57 | 11.12 | 6.9 |
| 43 | ELSDB02004 | SAF | *Episoriculus sacratus* | 18.11 | 3.39 | 8.58 | 6.2 | 1.41 | 7.78 | 7.75 | 7.77 | 4.53 | 11.01 | 6.98 |
| 44 | WWS12058 | SAF | *Episoriculus sacratus* | 18.1 | 3.41 | 8.94 | 6.1 | 1.44 | 7.83 | 7.78 | 7.7 | 4.55 | 11.11 | 6.9 |
| 45 | XZ13041 | SAF | *Episoriculus soluensis* | 17.9 | 3.65 | 9.25 | 5.74 | 1.69 | 8.08 | 7.99 | 7.77 | 4.87 | 12.33 | 7.52 |
| 46 | 77025 | KIZ | *Episoriculus soluensis* | 17.89 | 3.5 | 9.52 | 5.74 | 1.66 | 8.01 | 7.93 | 7.62 | 4.87 | 12.41 | 7.63 |
| 47 | ZHU032 | KIZ | *Episoriculus soluensis* | 17.9 | 3.66 | 9.21 | 5.81 | 1.77 | 8.11 | 8.01 | 7.91 | 4.88 | 12.22 | 7.34 |
| 48 | NJ13091 | SAF | *Episoriculus umbrinus* | 18.12 | 3.26 | 8.81 | 5.81 | 1.32 | 7.92 | 8.31 | 7.78 | 4.32 | 11.11 | 6.97 |
| 49 | 73996 | KIZ | *Episoriculus umbrinus* | 18.52 | 3.66 | 8.58 | 5.65 | 1.31 | 8.56 | 8.61 | 8.14 | 4.69 | 10.89 | 7.34 |
| 50 | 74018 | KIZ | *Episoriculus umbrinus* | 18.1 | 3.41 | 8.45 | 5.7 | 1.25 | 8.26 | 7.91 | 7.93 | 4.29 | 10.78 | 7.11 |
| 51 | 74059 | KIZ | *Episoriculus umbrinus* | 18.15 | 3.76 | 8.43 | 5.67 | 1.36 | 8.15 | 8.08 | 8.16 | 4.53 | 11.24 | 7.39 |
| 52 | 74064 | KIZ | *Episoriculus umbrinus* | 18.55 | 3.55 | 8.54 | 5.77 | 1.44 | 8.01 | 8.44 | 7.98 | 4.55 | 11.23 | 7.44 |
| 53 | 74080 | KIZ | *Episoriculus umbrinus* | 18.1 | 3.42 | 8.43 | 5.35 | 1.37 | 8.21 | 8.01 | 8.03 | 4.31 | 11.19 | 7.55 |
| Number | Sample ID | Museum | Species | CIL | IOB | CB | BH | MB | PIL | PPL | UTR | M^2^-M^2^ | ML | LTR |
| 54 | 74151 | KIZ | *Episoriculus umbrinus* | 18.58 | 3.5 | 8.45 | 5.53 | 1.44 | 8.35 | 8.01 | 8.22 | 4.35 | 11.21 | 7.63 |
| 55 | 74361 | KIZ | *Episoriculus umbrinus* | 18.11 | 3.71 | 8.45 | 5.61 | 1.37 | 8.31 | 7.91 | 8.05 | 4.46 | 11.47 | 7.66 |
| 56 | 77016 | KIZ | *Episoriculus umbrinus* | 18.35 | 3.44 | 8.48 | 5.57 | 1.48 | 8.11 | 7.86 | 8.18 | 4.62 | 11.49 | 7.45 |
