# Supplementary material for: Integrative phylogenetic analysis of the genus Episoriculus (Mammalia: Eulipotyphla: Soricidae)
Source: PLoS One. 2025 Jan 17;20(1):e0299624. doi: 10.1371/journal.pone.0299624 (PMC11981537; doi:10.1371/journal.pone.0299624)
Supplement: S2 Table — (DOCX) [file pone.0299624.s002.docx]

**S2 Table Results of ABGD species definition based on *CYTB* gene**

| relative gap width | Number of species  when P=0.021544 | Number of species  when P=0.035938 |
| --- | --- | --- |
| 0.5 | 10 | 9 |
| 0.75 | 9 | 9 |
| 1 | 9 | 9 |
| 1.25 | 9 | 9 |
