# Supplementary material for: Integrative phylogenetic analysis of the genus Episoriculus (Mammalia: Eulipotyphla: Soricidae)
Source: PLoS One. 2025 Jan 17;20(1):e0299624. doi: 10.1371/journal.pone.0299624 (PMC11981537; doi:10.1371/journal.pone.0299624)
Supplement: S3 Table — (DOCX) [file pone.0299624.s003.docx]

**S3 Table Posterior probabilities supporting three species (*Episoriculus caudatus*, *E. sacratus*, and *E. umbrinus*) as potential species using different algorithms and priors**

| **nDNA-algorithm0** | **Species delimitation** | 1 0 2 | 1 0 20 | 1 0 20 | 1 0 2 | 1 0 10 | 1 0 2= | Menn |
| --- | --- | --- | --- | --- | --- | --- | --- | --- |
|  |  | Heredity= 1 1 4 | Heredity= 1 1 4 | Heredity= 1 1 4 | Locustrate = 1 1 10 | Locustrate=1 1 10 | Locustrate=1 1 10 |  |
| *Episoriculus umbrinus*, *E. caudatus* | | 1 | 1 | 1 | 0.76312 | 1 | 1 | 1 |
| (*E. umbrinus*, *E. caudatus*), *E. sacratus* | | 1 | 1 | 1 | 1 | 1 | 1 | 1 |
| Inl (mean) | | -9713.536083 | -9713.503642 | -9713.595754 | -9714851074 | -9715.015544 | -9714.823643 |  |
| SD [InL (medium)] | | 4.398502 | 4.431084 | 4.374881 | 4572501 | 4.796982 | 4.567807 |  |
| **nDNA-algorithm1** | **Species delimitation** | 1 1 1.5 1 | 1 1 1 0.5 | 1 1 2 2 | 1 1 1.5 1 | 1 1 1 1.5 1 | 1 1 2 2 | Menn |
|  |  | Heredity= 1 1 4 | Heredity= 1 1 4 | Heredity= 1 1 4 | Locustrate = 1 1 10 | Locustrate=1 1 10 | Locustrate=1 1 10 |  |
| *E. umbrinus*, *E. caudatus* | | 1 | 1 | 1 | 1 | 1 | 1 | 1 |
| (*E. umbrinus*, *E. caudatus*), *E. sacratus* | | 1 | 1 | 1 | 1 | 1 | 1 | 1 |
| Inl (mean) | | -9713.608633 | -9713.623100 | -9713.550002 | -9714.892496 | -9714.880758 | -9714.769676 |  |
| SD [InL (medium)] | | 4.445452 | 4.491096 | 4.406825 | 4.643434 | 4.633969 | 4.558976 |  |
