# Supplementary material for: Integrative phylogenetic analysis of the genus Episoriculus (Mammalia: Eulipotyphla: Soricidae)
Source: PLoS One. 2025 Jan 17;20(1):e0299624. doi: 10.1371/journal.pone.0299624 (PMC11981537; doi:10.1371/journal.pone.0299624)
Supplement: Attachment — Submitted filename: Reply on comments from editors and Reviewer.docx [file pone.0299624.s004.docx]

**Reply on comments of Associate Editor and two anonymous reviewers**

**Dear Branislav T. Šiler, Ph.D.**

Thank you very much for your encourage and comments on our paper. Your comments are very useful for us to improve our manuscript. We have carefully revised our paper according to your comments. We hope this revision suitable for publication on PLOS ONE.

And here are the answers to your questions:

**1. Comments：**Coalescence-based methods should be revised as suggested by Reviewer #2. Concatenation of three nuclear genes should not be performed, mainly due to possible differences gene inheritance, which affect gene variation, and processing of alleles. The introduction section lacks flow, since gaps in the content are notified by Reviewer #1. Diversification of samples into two subgenera might not be supported. Constructing haplotype networks separately for each nuclear and a mitochondrial gene might point at different relationships.

**Reply:** In the manuscript, we used the nuclear gene connected dataset for phylogenetic analysis. The reason is that some species were not amplified on a certain gene, or the amplified sequences were less, so we also hope to include more species by concatenating multiple nuclear genes. Meanwhile, we also accepted you and the Reviewer 2's suggestion to conduct phylogenetic analysis based on single nuclear genes, and their tree structure is consistent with that of the nuclear gene connected dataset.

We also agreed with you and the Reviewers 1 that the classification of subgenera was premature, so we have removed this section from the manuscript to focus more on taxonomic issues between species.

**2. Comments:** Table 1: please add years of publications in parenthesis of authors' names.

**Reply:** We have added the publication to the corresponding author.

**3. Comments:** Table 2 should contain geographic coordinates of localities.

**Reply:** We have supplemented the latitude and longitude of the self-amplified sequences and collected information for this study.

**4. Comments：**Figures 2 & 3: please increase visibility of samples in the tree (possible font size increase or simply to use a wider font). What “Numbers at nodes.” means in the captions? Should the authors describe what they represent?

**Reply:** We re-edited the text and numbers in the Figure, and we also re-explained the information in the picture in detail in the legend.

**5. Comments:** Figures 4, 5, & 6 lack resolution. Please improve this and submit all figures as separate files.

**Reply:** We reworked figures 4,5, and 6 to improve the image resolution.

**6. Comments：**The text is abundant in randomly capitalized terms. Please avoid it. Generally, language usage should be considerably improved. The authors are advised to have the manuscript edited by a native English speaker or a professional editing agency.

**Reply:** We have revised the language to meet the requirements for publication.

Thanks again for your letter. We hope that we can accord with the publication requirements through this revision, and have the honor to publish our paper in PLOS ONE. And we will include all responses to the reviewer at the end of the letter.

best wishes!!

**Reply to two anonymous reviewers：**

Review Comments to the Author

Please use the space provided to explain your answers to the questions above. You may also include additional comments for the author, including concerns about dual publication, research ethics, or publication ethics. (Please upload your review as an attachment if it exceeds 20,000 characters)

Reviewer #1: Overall, this paper covers an interesting topic of differentiating lineages of shrews in China. It needs some improvements before it could be considered for publication, and I might suggest a more specialized journal like the Journal of Mammalogy, but with improvements it will be scientifically sound for PLoS One.

**Reviewer #1:** Overall, this paper covers an interesting topic of differentiating lineages of shrews in China. It needs some improvements before it could be considered for publication, and I might suggest a more specialized journal like the Journal of Mammalogy, but with improvements it will be scientifically sound for PLoS One.

Major comments

First, the introduction in particular could benefit from a native English speaker reading over it. It makes sense, but the sentences don't build on each other the way they should. There are many copy-editing changes that could be made, but I am not going to go into detail on those.

**Reply:** We have sorted out the relevant content, added some important information, and modified some language expressions.

Second, the way the Sanger Sequencing data are used and displayed isn't ideal. For the species-tree analyses, the nuclear loci should NOT be concatenated. They should be entered in as separate genes into *BEAST, and the authors must make sure to designate the mode of inheritance (mitochondrial vs. nuclear). It was not clear in the manuscript if this was done.

**Reply:** In the manuscript, we used the nuclear gene connected dataset for phylogenetic analysis. The reason is that some species were not amplified on a certain gene, or the amplified sequences were less, so we also hope to better included more species to the phylogeny by concatenating multiple nuclear genes. Meanwhile, we also accepted your suggestion to conduct phylogenetic analysis based on single nuclear genes, and obtained tree structure consistent with that of the nuclear gene connected dataset.

Third, I would recommend that the authors explore the gene trees as haplotype networks instead. It's possible that some of the problems with comparing the nuclear and mitochondrial loci are due to forcing a bifurcating tree on a non-bifurcating dataset. I don't know if this would help, but it would be quick and interesting to explore (the program popART will do this easily).

**Reply:** The sequences currently used are ones that we have already picked out using haplotype calculations. At the same time, we confirm that the number of some species is too small, which will cause some species to be missing from the phylogenetic tree or affect the topology of the phylogenetic tree.

Fourth, I don’t think there is justification for two subgenera. These seem fine within one genus and subgenera appear to be over-splitting.

**Reply:** We agree with your opinion. We have deleted part of the content in the article.

Specific responses:

General intro: doesn’t read very smoothly and could use more copy editing.

Line 17: not clear how this is different from the previous sentence.

**Reply:** We have revised the sentence.

Line 48: Did this cover all of the proposed subspecies? Make the sampling compared to the subspecies clear here (it’s clearer later that some groups are missing).

**Reply:** We have supplemented this part in the section of method. In this study, we don‘t pay too much attention to the classification of subspecies, but only discuss in detail the subspecies of a certain species that is more controversial.

Line 50: this is too detailed for the intro. More on why this is important.

**Reply:** We feel that this section is superfluous and we have moved it to the section of Methods

Line 93: these should not be concatenated. These should be run in StarBEAST in the BEAST2 package. That is very fast and easy to run, and more informative/correct than concatenated data. It can also scale the inheritance for mtDNA and nuclear, which you need for this dataset, and can allow all individuals within a population to represent that species together.

**Reply:** We considered that nuclear genes connected dataset could cover more species, and we agree with you that we complement phylogenetic trees based on single nuclear genes.

Line 94: each nuclear gene needs it own assessment in jModelTest

**Reply:** We have revised and supplemented this content.

Line 96: Neogale vision is not mentioned as part of this dataset. Is this from genbank? This all needs to be clearer.

**Reply:** We have corrected this error.

Line 98: concatenated

**Reply:** We have corrected this error.

Line 100: below

**Reply:** We have corrected this error.

Line 118: were males and females evaluated separately?

**Reply:** Through the study of the related species of Episoriculus, we didn’t find that the existence of sexual duality has an impact on taxonomic research. Therefore, we only select adult individuals with intact skull.

Line 134: Change “once” to “was”

**Reply:** We agree with you and have revised it in the manuscript.

Line 135: full genus at the start of a sentence.

**Reply:** We agree with you and have revised all these problems in the manuscript.

Line 144: these should not be concatenated. I think either 4 gene trees or 4 haplotype networks should be shown for this figure.

Reply: We agree with you. We put the tree base on the *CYTB* and the tree based on the concatenated nuclear gene on one figure, and the tree of three separate nuclear genes on another figture.

Line 162: remove italics on “clustered”

**Reply:** We have corrected this error.

Line 181: "usually" isn't informative. Is this the mean? Median?

**Reply:** We agree with you and have revised it in the manuscript.

Line 194: PC1?

**Reply:** We have corrected this error, and replaced PC1 with PC 1.

Line 196: it's PC1, so of course it has the highest degree

**Reply:** We agree with you and have revised it in the manuscript.

Line 201: reframe

**Reply:** We agree with you and have revised it in the manuscript.

Line 203: all of this can be simplified to say that the three species are non-distinguishable while the other three are. Could do a DFA.

**Reply:** We re-analyzed the measured data of the species and concluded that pca analysis could complete the statistical analysis of the skull.

Line 214: remove “very”

**Reply:** We have removed it.

Line 216: is this based on PCA loadings or separate analyses?

**Reply:** This part is based solely on morphological comparison.

Line 223: lowercase “ancient”

**Reply:** We have corrected it.

Line 231: this isn’t necessarily the right way to do it. Genomics seems better in this case than mtDNA and sanger nuclear.

**Reply:** We agree with your opinion. Based on current phylogenetic studies of existing related species, mitochondria and partial nuclear genes can also be analyzed with good results. Of course, future studies of Episoriculus should be conducted at a deeper level, such as the genomics level, and we consider that more information about phylogeny will be obtained.

Line 266: the systematic account is too short. It should contain more comparisons to other species and the spatial distribution (which is part of the description in this case).

**Reply:** We have removed this section. We agree with you that the subgeneric division is excessive.

Line 280: I don’t think there is justification for two subgenera. These seem fine within one genus and subgenera appear to be over-splitting.

**Reply:** We agree with your opinion and have removed this section.

Table 1: these should have the years, so we can better see the progression. Maybe an indicator of a newly discovered species vs. a "split" species

**Reply:** We have added the author's corresponding year in Table 1.

Fig 1: what does this mean that it's the figure 2 legend?

Could this figure also contain polygons for where the two other species (not included) would be found?

**Reply:** We have corrected the wrong legend. And in Fig.1, we only displayed the species we collected, *Episoriculus baileyi* and *Pseudosoriculus* were not collected, so they were not displayed in the figure.

Figure 3: mistake. The bottom clade are caudatus

**Reply:** We agree with you and have revised it in the figure.

**Fig. 4 and 5: figures 5 and 6 should be combined**

**Reply:** We have revised all figures and removed the original figure 6.

Figure 7: the drawings should be comparable. One is black and shaded and one is a while outline. This artificially makes them look more different.

**Reply:** We have revised this content and repainted the teeth.

**Reviewer #2**: Review of Molecular and morphological analyses of the genus Episoriculus with the description of a new subgenus.

The present study uses a combination of molecular and morphological data to evaluate the taxonomic status of a number of members of the genus Episoriculus and its relatives. There is quite of lot of valuable information here, but as outlined below, I have a large number of small editorial suggestions, plus a few more substantive suggestions for additional analyses that I believe will strengthen the paper.

l. 2. Replace “It occurs…” with “The genus Episoriculus occurs…”

**Reply:** We have revised it.

l. 16. Replace “believe” with “suggest” or “maintain”.

**Reply:** We have revised it.

l. 17. Replace “While” with “However,”

**Reply:** We have revised it.

l. 19. Replace “And” with “In addition”.

**Reply:** We deleted this sentence from the manuscript.

l. 45. Replace “Wison” with “Wilson”

**Reply:** We have revised the above statement in accordance with your comments

l. 50. Modify this to read “We amplified a portion of a mitochondrial gene (Cyt b)…” [I assume that you only looked a portion of each of these gene as opposed to the complete genes. IF you did sequence the entire gene, then leave the sentence as it is.]

**Reply**: In this study, we amplified only one mitochondrial gene. We sequenced only one gene segment in the mitochondria and did not measure the entire gene segment.

l. 74. Perhaps specify the concentration of the primers used.

**Reply**: We do not think it is necessary to explain this, so we have not added.

l. 68-69. The text should say, “This is the Fig. 1 legend.”

**Reply:** We have corrected this error.

l. 88-89. This is a bit confusing. If ALL sequences, both mitochondrial and nuclear were aligned and examined in MEGA, then start by saying that. Then state that screening for heterozygous positions was also performed in MEGA for nuclear genes. Mitochondrial genes only come from the female parent, and so typically are homoplasmic. Because mitochondrial DNA is haploid, the term heterozygous does not apply to it.

**Reply:** We have revised this content. We also considered that ‘mitochondrial DNA is haploid, the term heterozygous does not apply to it’ is inaccurate, so we have revised this content

L. 89- 90. This is confusing as written. Re-write.

**Reply:** We have revised this content.

l. 96. Neogale vison should be italicized.

**Reply:** We have corrected this error.

l. 98-100. Concate-nated and be-low do not need hyphens in them.

**Reply:** We have corrected this error.

In Figure 2, macrurus is misspelled. Also, SP. should be sp.\

**Reply:** We have corrected this error.

l. 102 to 109. I think that the analysis would be strengthened considerably by using one of the following coalescence-based methods:

Puillandre N, Lambert A, Brouillet S, Achaz G. ABGD, Automatic Barcode Gap Discovery for primary species delimitation. Molecular ecology. 2012; 21(8):1864–77.

Pons J, Barraclough TG, Gomez-Zurita J, Cardoso A, Duran DP, Hazell S, et al. Sequence-based species delimitation for the DNA taxonomy of undescribed insects. Systematic biology. 2006; 55 (4):595–609.

Monaghan MT, Wild R, Elliot M, Fujisawa T, Balke M, Inward DJ, et al. Accelerated species inventory on Madagascar using coalescent-based models of species delineation. Systematic biology. 2009; 58(3):298–311.

**Reply:** To enrich the results of species definition, we have supplemented the analysis of ABGD, GMYC and BPP in the manuscript.

l. 113. Avoid starting a sentence with a number (in this case 56). Instead say something like, “A total of 56 complete skulls…”.

**Reply:** We agree with you and have revised it. We supplemented the analysis of ABGD, GMYC and BPP in the species definition.

l. 124. Provide a bit more information on why the KMO and Bartlett’s sphere tests were performed. What would you do if there were correlations or partial correlations between variables or “if the correlation matrix was the unit matrix and the independent analysis method of each variable was invalid”. In particular, I do not understand the significance of the results of the Bartlett’s sphere test.

**Reply**: Before analyzing the measured data, we perform a KMO test to study the partial correlation coefficient of the variable and determine whether the variable is suitable for factor analysis. At the same time, we also perform Bartlett’s test of sphericity to determine that the correlation matrix is not an identity matrix, meaning that the variables are highly correlated enough to provide a reasonable basis for factor analysis. If these two tests are not met, we will perform a T-test on the variable.

L. 130. I think this should just say “Results” (not “Results and discussion”). There is a separate section called “Discussion” starting on l. 219, and I think this section from 130 to 219 is just Results.

**Reply:** We have reorganized the content of the Results chapter and moved some of the content into the Discussion chapter.

l. 133. I would add the word “putative” here. Without further discussion, these should be considered putative or possible species. And technically, there are more monophyletic groups within each of the clades that you identify. My point saying that is that it is not only the phylogenetic topology that will determine what is a species, but also the amount of average sequence divergence between clades.

**Reply:** We agree with you and have revised it in the manuscript.

L. 134…that once WAS part of …

**Reply:** We considered there's a problem with that statement, and have revised it in the manuscript.

L. 163. Not clear what the authors are saying about E. sacratus and Episoriculus sp. here.

**Reply:** We considered there's a problem with that statement, and have revised it in the manuscript

l. 194. Change to, “The factor loading values for PC1 were all positive…” I do not see where the loading values are above 0.95? They seem to range from 0.030 to 0.888. Am I missing something here? You say “the features with factor load > 0.9 included PIL, ML, CIL, UTR, and LTR.” However, none of these values in the table are above 0.9.

**Reply:** We agree with you and have revised it in the manuscript.

l. 200-201. I do not understand this sentence. Clarify your meaning here. I see that E. leucops, E. macrurus, and E. soluensis form discrete clusters, primarily separated from one another along PC2, but that is not what this sentence says.

**Reply:** We agree with you and have revised it in the manuscript.

l. 206. I don’t believe that the methodology of the LAD maps is presented anywhere.

**Reply:** We removed the LAD.

l. 206. Generally, I don't think that it is appropriate to say that this or that specimen was wrongly classified necessarily simply based on morphology. There could be cryptic species involved. What do the molecular data say for these particular specimens (or perhaos you do not have those data for these particular samples).

**Reply:** The samples that we use morphologically include all the species that we can collect, and correspond molecularly to each other. When we discuss the classification of species, we need to combine molecular and morphological data for analysis.

l. 222-223. I don't know what you mean by differences in genetic background. Also, Ancient should be ancient (no capital A). Also, how could “burst formula speciation” lead to this pattern? Also, why is Formula capitalized on line 224. This sentence is simply too vague to be useful. Each of these possible explanations should be developed a bit further.

**Reply:** We combed through the text again.

l. 226 following. I have some questions regarding the analysis and interpretation. Why was C. fuliginosa chosen as the outgroup when there is only one nuclear gene available for that taxon? Also, maybe redo the Cyt b tree without C. fuliginosa. Maybe include some additional outgroup taxa. There are several nodes (branches) that have less than 0.95 pp. This lack of resolution means that you cannot have strong confidence in the basal relationships in the tree and therefore no strong confidence for where the E. macrurus clade really occurs in the tree. Much of the basal part of the tree for cyt b should be considered an unresolved polytomy.

**Reply:** We selected only a sample of *Crocidura* as an outgroup, but because it is far related to Soricinae. We confirm that it can also have a significant impact on the structure of the system tree. In order to better showing the taxonomic position of the spcies of *Episoriculus* and *Pseudosoriculus*., we also supplemented the sequences of other genera of Soricinae for analysis to better improve the structure of the system tree.

L. 259. There is not sufficient discussion of the value of the Kimura’s two parameter distances. Is there an acceptable threshold value for K2P divergence that is likely indicative of species level divergence? What do shrew studies in general say about a cyt b divergence value that is likely indicative of a “good species”?

**Reply:** Bradley (2001) considered that genetic distance values ,2% were indicative of intraspecific variation; values between 2 and 11% had a high probability of being indicative of conspecific populations or valid species and merit additional study concerning specific status; and values .11% were indicative of specific recognition. Based on this point of view, we discuss species classification in terms of genetic distance. But we also need to fully integrate the result of phylogenetic and morphological to determine the validity of species.

l. 270. For the proposed Genus name, Longi is used more typically, I think, than Longa when combined with cauda, e.g., Longicauda.

**Reply:** We have eliminated the discussion of subgenera, so we do not refer to the naming of subgenera in the text.

l. 282. Two subgenera (vs. two subgenus). Subgenera is the plural of subgenus.

**Reply:** We believe that the classification of subgenera is redundant, so we have removed the content about subgenera. Therefore, we will not modify the above two problems in the article.
