# Supplementary material for: Integrative phylogenetic analysis of the genus Episoriculus (Mammalia: Eulipotyphla: Soricidae)
Source: PLoS One. 2025 Jan 17;20(1):e0299624. doi: 10.1371/journal.pone.0299624 (PMC11981537; doi:10.1371/journal.pone.0299624)
Supplement: Attachment — Submitted filename: Response.docx [file pone.0299624.s006.docx]

Dear editor,

We are grateful for your email and suggestions for our manuscript "Integrative phylogenetic analysis of the genus Episoriculus (Mammalia: Eulipotyphla: Soricidae) " (PONE-D-24-05960R1). Following the comments, we have made the corrections accordingly and highlighted the revised portion in blue in the manuscript.

Best regards,

Liu Yingxun

**ASSOCIATE EDITOR COMMENTS:**

1.When completing the data availability statement of the submission form, you indicated that you will make your data available on acceptance. We strongly recommend all authors decide on a data sharing plan before acceptance, as the process can be lengthy and hold up publication timelines. Please note that, though access restrictions are acceptable now, your entire data will need to be made freely accessible if your manuscript is accepted for publication. This policy applies to all data except where public deposition would breach compliance with the protocol approved by your research ethics board. If you are unable to adhere to our open data policy, please kindly revise your statement to explain your reasoning and we will seek the editor's input on an exemption. Please be assured that, once you have provided your new statement, the assessment of your exemption will not hold up the peer review process.

Response: We have already submitted all the data and made public. Additionally, we have added the related description at line 191-194.

2. Please ensure that you refer to Table 4 in your text as, if accepted, production will need this reference to link the reader to the Table
Response: Thanks for spotting this. We revised this issue and added the reference at line 100.

3. To comply with PLOS ONE submissions requirements, in your Methods section, please provide additional information regarding the experiments involving animals and ensure you have included details on (1) methods of sacrifice, (2) methods of anesthesia and/or analgesia, and (3) efforts to alleviate suffering

Response: We reformulated our description of the methods section, and provided the additional information at line 65-72.
