# Supplementary material for: Integrative phylogenetic analysis of the genus Episoriculus (Mammalia: Eulipotyphla: Soricidae)
Source: PLoS One. 2025 Jan 17;20(1):e0299624. doi: 10.1371/journal.pone.0299624 (PMC11981537; doi:10.1371/journal.pone.0299624)
Supplement: Attachment — Submitted filename: Response.docx [file pone.0299624.s007.docx]

Dear Branislav T. Šiler, Ph.D.,

We are grateful for your email and suggestions for our manuscript "Integrative phylogenetic analysis of the genus *Episoriculus* (Mammalia: Eulipotyphla: Soricidae) " (PONE-D-24-05960R1). Following your comments, we have made the corrections accordingly in the manuscript.

We have re-uploaded figure 1 in compliance with copyright regulations. We uploaded the CC BY 4.0 license file, in which we obtained the permission and signature of the image owner. We have made some revisions to the references. If the permission is not approved, we considered that it is feasible to delete Figure 1, which will not reduce the quality of the manuscript, avoid copyright disputes, and speed up the review progress. And we confirm that laboratory protocol is not suitable for our study, because there is uniqueness in sampling, and other research groups cannot collect the same samples. But we also submitted a laboratory protocol in other section according to the format for reference.

We hope that we can meet the requirements of modification this time.

Best regards,

Liu Yingxun

**ASSOCIATE EDITOR COMMENTS:**

1. I find the reviewers' concerns fairly addressed, as the phylogenetic relationships have been recalculated and the text amended according to their suggestions. In their revision, the authors have left brackets in L65 empty: L65: "After euthanasia with eugenol, following the ASM guidelines ()".

Please fill the brackets with the citation and provide the reference in the final list.

**Response**: We have filled the brackets with the citation and added the reference in the final list.

2. We note that [Figure 1] in your submission contain [map/satellite] images which may be copyrighted. All PLOS content is published under the Creative Commons Attribution License (CC BY 4.0), which means that the manuscript, images, and Supporting Information files will be freely available online, and any third party is permitted to access, download, copy, distribute, and use these materials in any way, even commercially, with proper attribution. For these reasons, we cannot publish previously copyrighted maps or satellite images created using proprietary data, such as Google software (Google Maps, Street View, and Earth). For more information, see our copyright guidelines: http://journals.plos.org/plosone/s/licenses-and-copyright.

**Response**: We have re-uploaded figure 1 in compliance with copyright regulations.
We also uploaded the CC BY 4.0 license file, in which we obtained the permission and signature of the image owner.

3. Please review your reference list to ensure that it is complete and correct. If you have cited papers that have been retracted, please include the rationale for doing so in the manuscript text, or remove these references and replace them with relevant current references. Any changes to the reference list should be mentioned in the rebuttal letter that accompanies your revised manuscript. If you need to cite a retracted article, indicate the article’s retracted status in the References list and also include a citation and full reference for the retraction notice.

**Response**: We have made some revisions to the references.

**Journal Requirements:**

1. Please review your reference list to ensure that it is complete and correct. If you have cited papers that have been retracted, please include the rationale for doing so in the manuscript text, or remove these references and replace them with relevant current references. Any changes to the reference list should be mentioned in the rebuttal letter that accompanies your revised manuscript. If you need to cite a retracted article, indicate the article’s retracted status in the References list and also include a citation and full reference for the retraction notice.

Response: We have reviewed the references, and determined that no references have been withdrawn or unpublished.

2. While revising your submission, please upload your figure files to the Preflight Analysis and Conversion Engine (PACE) digital diagnostic tool, https://pacev2.apexcovantage.com/. PACE helps ensure that figures meet PLOS requirements. To use PACE, you must first register as a user. Registration is free. Then, login and navigate to the UPLOAD tab, where you will find detailed instructions on how to use the tool. If you encounter any issues or have any questions when using PACE, please email PLOS at <a href="mailto:figures@plos.org">figures@plos.org. Please note that Supporting Information files do not need this step.

Response: We have uploaded and archived the six figures to PACE and modified it as required.
